# Supplementary material for: Role of cell-type specific nucleosome positioning in inducible activation of mammalian promoters
Source: Nat Commun. 2020 Feb 26;11:1075. doi: 10.1038/s41467-020-14950-5 (PMC7044431; doi:10.1038/s41467-020-14950-5)
Supplement: Supplementary file 2 — Supplementary Information [file 41467_2020_14950_MOESM2_ESM.pdf]

Role of cell-type specific nucleosome positioning in inducible activation of mammalian promoters

Agata Oruba[1], Simona Saccani[2,1,3] & Dominic van Essen[2,1,3]

[1] Max Planck Institute for Immunobiology & Epigenetics, Stübeweg 51, Freiburg D79108, Germany
[2] Institute for Research on Cancer & Aging, Nice (IRCAN), 28 Avenue Valombrose, Nice 06107, France
[3] Equal contributions & corresponding authors: dvanessen@unice.fr; ssaccani@unice.fr

Supplementary figures

Supplementary figure 1

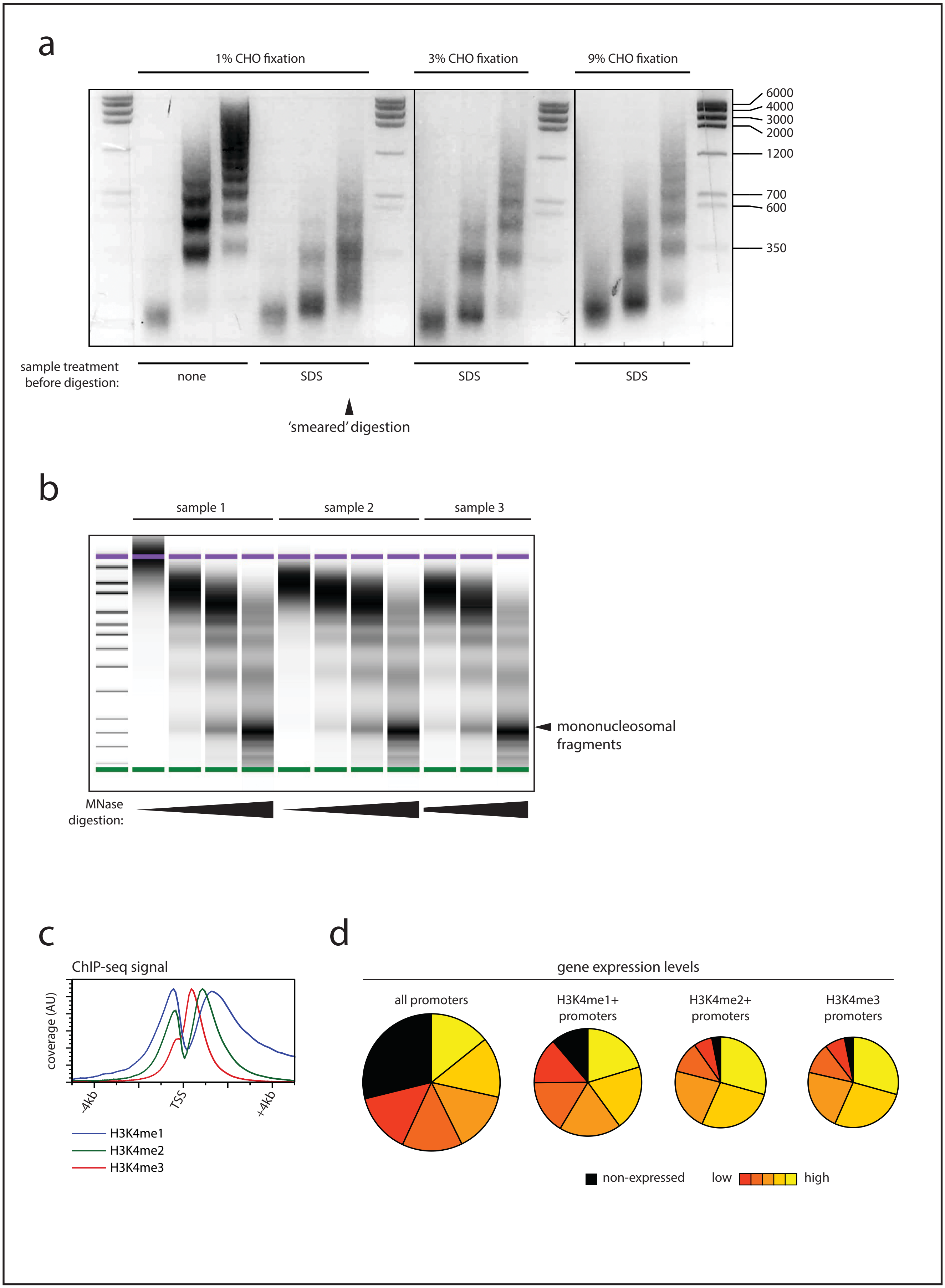

Supplementary figure 1

a: Strong crosslinking requirement to immobilize nucleosomes at *in vivo* positions

Agarose gel electrophoresis of DNA fragments produced by MNase digestion of samples prepared using different fixation conditions and treatments: in this series of pilot experiments, we observed that insufficient cross-linking can allow ‘smearing’ of the characteristic nucleosomal ladder of MNase-digested chromatin, particularly when samples were exposed to conditions typically used during ChIP, implying that strong fixation is required to confidently ensure that the pattern of DNA protection measured *in vitro* indeed reflects the original *in vivo* nucleosome positioning.
Cells were cross-linked using mild (1% formaldehyde; left panel), strong (3% formaldehyde, middle panel) or very strong (9% formaldehyde; right panel) conditions, and chromatin was prepared using either non-denaturing conditions (left panel, lanes 2-4), or using conventional ChIP buffers including SDS (other lanes). Each group of 3 lanes represent DNA fragments resulting from digestion with 50, 5 and 0.5 units of MNase. Mildly-crosslinked samples exhibit a clearly-defined nucleosomal ‘ladder’ under native conditions, but the fragment sizes appear ‘smeared’ when exposed to SDS (lane 7, indicated). This effect is reduced or abolished at higher levels of crosslinking (middle & right panels, 3rd lanes). This phenomenon highlights that nucleosomes on non-crosslinked chromatin are potentially free to move or partially unwind and allow MNase cleavage at normally-protected sites, which would lead to mistakenly inferred nucleosome positions based on digestion sites. Throughout this study, cells were fixed using 4% formaldehyde to immobilize nucleosomes at their *in vivo* positions, and no SDS treatment was used during ChIP.
Note that the increased presence of high molecular-weight fragments in natively-prepared chromatin samples is due to the reduced efficiency of initial fragmentation by sonication.

b: Unchanged mononucleosomal fragment size across a range of digestion levels

Capillary electrophoresis of representative examples of ChIP-MNase samples prepared using varying levels of MNase digestion. Lane 1: molecular size markers; lanes 2 & 6: non-MNase-digested H3K4me1-ChIP DNA (note the high mean fragment size range of approximately 3-10kb used for ChIP-MNase); lanes 3-5, 7-9 & 10-12: titrated on-beads digestion of chromatin after ChIP, using 0.25, 1.25 and 2.5 units of MNase. The same titration was performed for all ChIP-MNase samples in this study, and digested samples with approximately 80% mononucleosomal fragments (indicated) were used for sequencing and analysis (corresponding to digestion with 2.5units MNase for each of the 3 samples depicted here). Note that within this range of digestion levels, the apparent size of mononucleosomal DNA fragments is largely unchanged, with only a low amount of sub-nucleosomal fragments appearing at the highest MNase dose used: thus, under the fixation (4% formaldehyde) and sample preparation conditions used, we do not detect significant nucleosome unwinding/movement or digestion within the nucleosome footprint.

c,d: H3K4me1 marks active and inactive promoters

c. Mean profiles of H3K4me1 (blue[89]), H3K4me2 (green[90]) and H3K4me3 (red[89]) ChIP-seq coverage surrounding all promoters in fibroblasts. The broad profile of H3K4me1 makes it suitable as a mark to isolate promoters together with their upstream and downstream flanks. In addition, the progressively narrowing of the genomic regions that are marked by increasing levels of H3K4 methylation indicates that the broad H3K4me1 coverage at promoters cannot generally be lost during gene activation by hypermethylation to H3K4me2 or H3K4me3.
d. Proportion of promoters of non-expressed and expressed genes among all promoters (left) or among those represented within the upper 50% of total ChIP-seq coverage for H3K4me1 (left centre), H3K4me2 (right centre) or H3K4me3 (right), in fibroblasts. Expressed genes are divided into 5 equal quantiles indicated by colour. The size of each pie-chart is proportional to the fraction of all promoters represented within the upper 50% of total ChIP-seq coverage in each case. Note that H3K4me2 and H3K4me3 strongly enrich

for promoters of expressed genes, and among these they strongly enrich for those of the most highly-expressed genes. H3K4me1 exhibits much higher coverage at inactive promoters, and does not enrich to a large extent for promoters of highly-expressed genes.

Supplementary figure 2

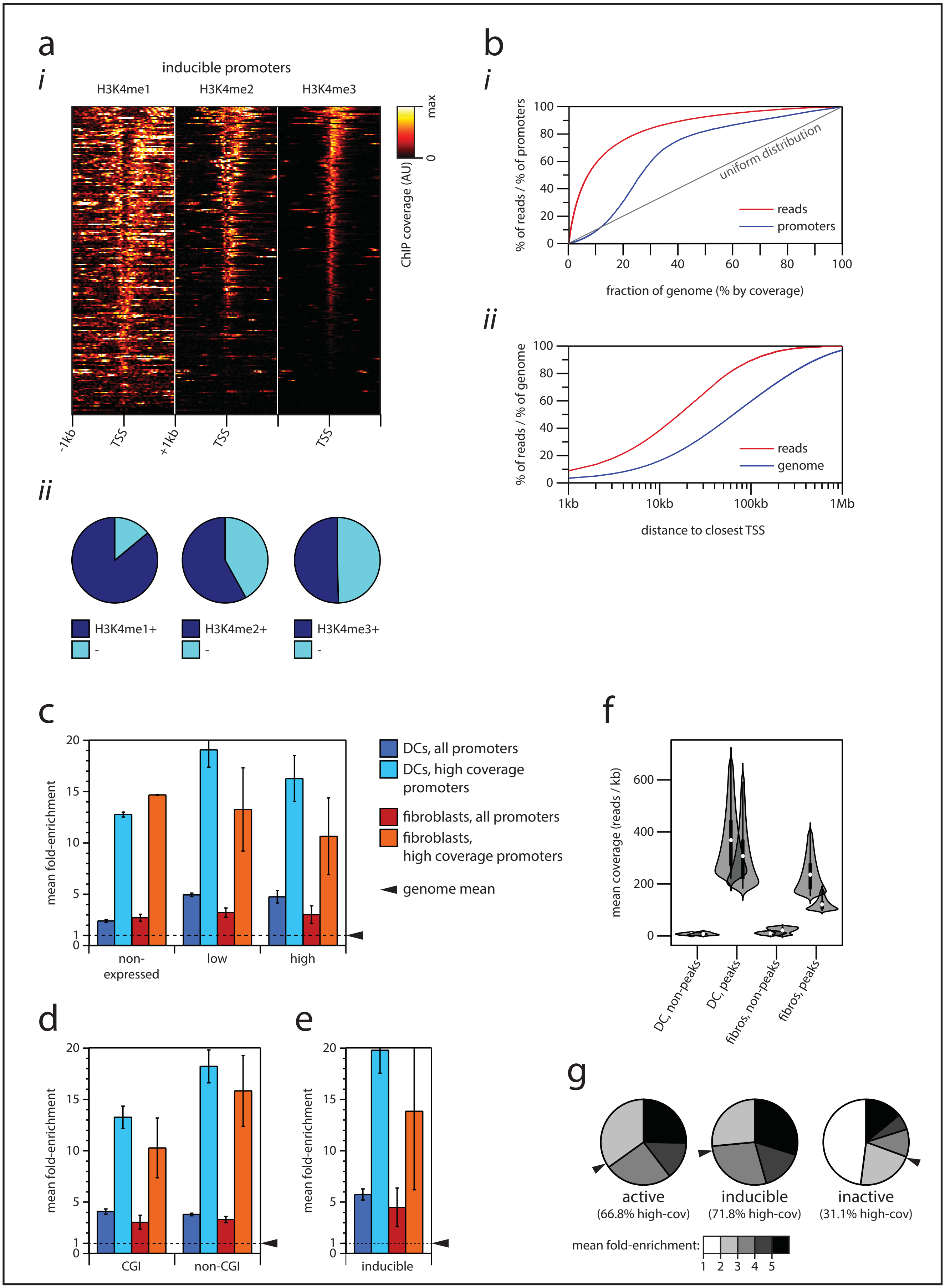

Supplementary figure 2

a: H3K4me1 marks inducible promoters

i. Heatmaps of ChIP coverage of H3K4me1 (left), H3K4me2 (middle) & H3K4me3 (right) surrounding inducible promoters in fibroblasts. ii. Fraction of inducible promoters that are represented within (blue) or absent from (cyan) the upper 50% of total ChIP-seq coverage for H3K4me1 (left), H3K4me2 (middle) and H3K4me3 (right). Note that only H3K4me1 exhibits high coverage across the majority of inducible promoters.

b: H3K4me1 ChIP-MNase enriches for a specific fraction of the genome including promoter-proximal regions

Cumulative fractions of ChIP-MNase sequencing reads (i and ii), fractions of annotated promoters (i) or fractions of the genome (ii) contained within a specified fraction of the genome defined by decreasing coverage (i), or within a specified distance to the closest annotated TSS (ii). 50% of sequence reads or of promoters (y-axis, panel i) fall within the highest-coverage 6.9% or 26.1% of the genome (x-axis, panel i), respectively. 39.1% of sequence reads (y-axis, panel ii) fall within 10kb of an annotated TSS (x-axis, panel ii), compared to 16.3% of the genome. The straight line in panel i ('uniform distribution') indicates the cumulative fraction of sequencing reads expected from uniform (non-enriched) distribution across the genome.

c-e: Highly-enriched sequence coverage by H3K4me1 ChIP-MNase at different classes of promoters

Fold-enrichment of sequence coverage by H3K4me1 ChIP-MNase within 2kb regions surrounding different promoter classes, compared to the level corresponding to uniform genomic coverage ('genome mean'; indicated as 1-fold). Blue: DCs, all promoters; Cyan: DCs, highest-coverage 10% of promoters; Red: fibroblasts, all promoters; Orange: fibroblasts, highest-coverage 10% of promoters. Promoters are divided into classes corresponding to those of non-expressed, low-expressd and high-expressed genes (panel c), CGI-containing and non-CGI containing promoters (panel d), or inducible promoters (panel e). Coverages of all promoter classes analysed are significantly enriched by H3K4me1 ChIP-MNase, both consdering all promoters (mean enrichments of 2.4- to 5.7-fold) and considering the highest-coverage promoters (mean enrichments of 8.4- to 15-fold). Source data are provided as a source data file.

f: ChIP specificity measured by coverage at peak regions

Distributions of coverage levels at ChIP peak regions (defined as the genomic intervals with the 5% highest coverage, excluding the top 0.5% [that may contain possible alignment or genome assembly artefacts]) and non-peak regions (defined as all genomic intervals with less than median coverage), in replicate samples of H3K4me1 ChIP-MNase in DCs and fibroblasts. Thick bars indicate limits of quartiles; dots indicate means.

g: Proportions of promoter sets used for analysis

Pie-charts depicting the proportions of active (i), inducible (ii) or inactive (iii) promoters in DCs, with mean fold-enrichments by H3K4me1 ChIP-MNase that exceed the indicated levels (white: mean enrichment greater than 1-fold; black: mean enrichment greater than 5-fold, compared to uniform genomic coverage). Arrowheads indicate the fraction of promoters belonging to each set that were used for analysis in this study (labelled below each pie).

Supplementary figure 3

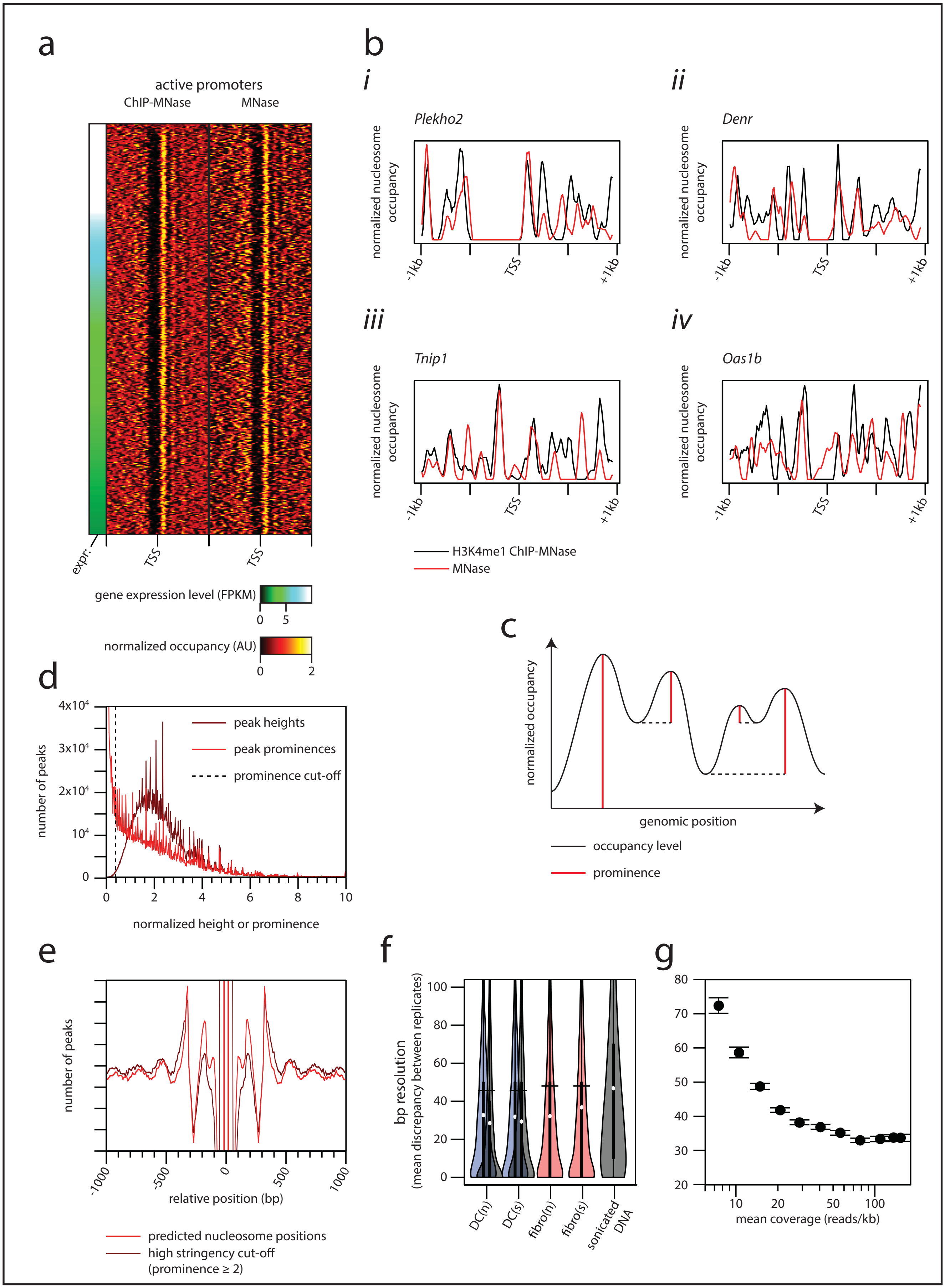

Supplementary figure 3

a,b: Comparison of H3K4me1 ChIP-MNase and whole-genome MNase at example promoters

a. Heatmap of nucleosome occupancy levels surrounding active promoters in fibroblasts, measured by H3K4me1 ChIP-MNase-seq, or by whole-genome MNase-seq; sidebar indicates corresponding gene expression levels.
b. Normalized nucleosome occupancy profiles in non-stimulated fibroblasts across individual promoters of the *Plekho2* (i; expressed in fibroblasts), *Denr* (ii; expressed in fibroblasts), *Tnip1* (iii; inducible in fibroblasts) and *Oas1b* (iv; non-expressed in fibroblasts) genes, measured by H3K4me1 ChIP-MNase-seq, or by whole-genome MNase-seq. Whole-genome MNase-seq data is pooled from[91]). Note that the level of coverage mapping to promoters in the whole-genome MNase-seq data analysed here (27 reads kb^{-1}) is lower than the level attained by ChIP-MNase (≥50 reads kb^{-1} at all promoters analysed), despite a substantially higher total sequencing depth (whole-genome MNase-seq: 192M total sequenced fragments; ChIP-MNase-seq: 37-40M sequenced fragments per replicate). Normalized occupancy levels across 10bp bins at TSS ±1kb measured by H3K4me1 ChIP-MNase and by genome-wide MNase-seq are correlated with Pearson's r=0.27 ($p=3.0\times10^{-7091}$).

c-e: Prediction of nucleosome positions by topographic prominence

c. Cartoon illustrating prominences of example peaks. Dotted lines indicate the lowest valley separating each peak from an adjacent higher peak (the 'key col'); red lines indicate the prominence of each peak (the height above the key col). Note that the prominence of each peak is not directly related to its height, and that minor perturbations in the profile typically have low prominence, even if the height may be large at that position: see also figure 1e.
d. Distributions of heights (maroon) and prominences (red) of all peaks defined as local maxima in normalized ChIP-MNase data from non-stimulated DCs, with the cut-off used to exclude measurement noise indicated (dotted line). 20% of peaks with prominences below the cut-off are excluded from predicted nucleosome positions, whereas less than 0.02% of peaks have heights below this level.
e. Distributions of relative distances for predicted nucleosome positions used in this study (red; corresponding to the prominence cut-off illustrated in panel d), or for high-stringency predicted positions (maroon; using a prominence cut-off of 2). Both distributions display a strongly phased pattern, with a period matching to the mean inter-nucleosome spacing. Note that high-stringency prediction excludes less-favoured alternative positions at shorter relative distances (small peaks detectable at relative position ±90bp).

f,g: Estimated resolution of predicted nucleosome positions

f. Resolution of predicted nucleosome positions, calculated as the mean discrepancy between replicate samples as in figure 1f. Blue violins: observed resolution of DC datasets; red violins: observed resolution of fibroblast datasets; grey violins ('DC[n]' and 'DC[s]'): resolution simulated by random sampling to eliminate biological variation; grey violins ('sonicated DNA') observed discrepancies calculated between mock nucleosome predictions using replicate samples prepared from randomly-fragmented (sonicated) DNA; lines: expected resolution based on random placement with matched mean density. Thick bars indicate limits of quartiles; dots indicate means.
g. Resolution of predicted nucleosome positions at different levels of local sequence coverage. Nucleosome positions were calculated at sampled genomic locations with different selected ranges of mean coverage, and the resolution at each sample was determined separately. Note that the discrepancy between replicates rises when mean coverage is below approximately 30 reads per kb. Mean coverage levels at promoters from samples in this study were 53 reads/kb (DCs) or 37 reads/kb (fibroblasts), and only promoters with ≥50 reads/kb were used for analysis (corresponding to 53% of all genomic

annotated promoters). Error bars indicate standard error of the mean (SEM). Source data are provided as a source data file.

Supplementary figure 4

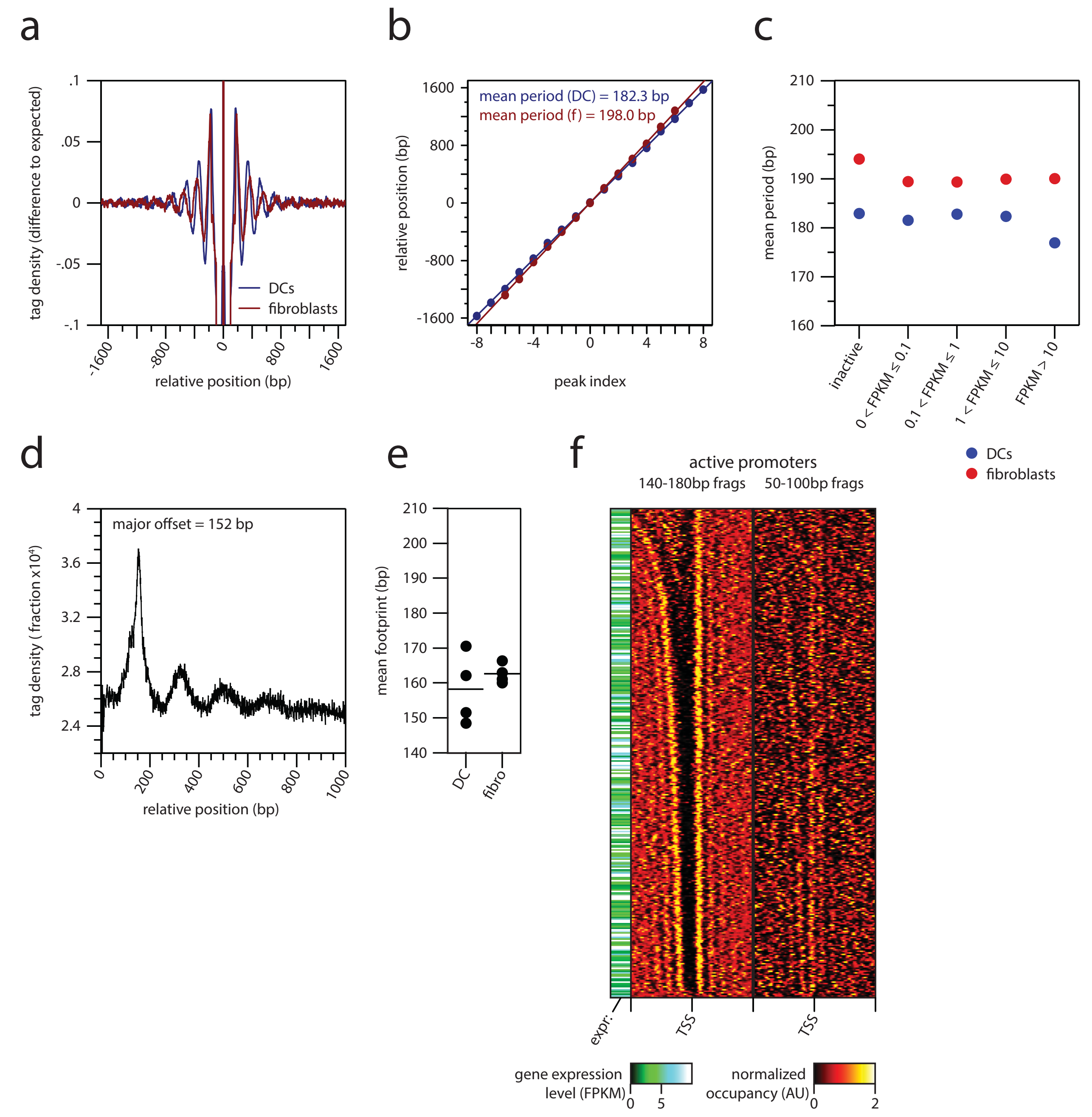

Supplementary figure 4

a-e: Periodicity of ChIP-MNase tags and mean nucleosome spacing

a. Example distributions of genomic distances between locations of nearby ChIP-MNase digestion fragment ends that are mapped on the same strand, in DCs (blue) and fibroblasts (red). Tag density is reported as the fractional difference to the level expected from uniform recovery across sliding 200bp windows.
b. Relative positions of local peaks in the distributions shown in panel a. Line is linear fit, with gradient corresponding to the mean inter-peak (and hence inter-nucleosomal) period. Coefficient of determination: DC: R^2=0.9997, fibroblasts: R^2=0.9996.
c. Mean nucleosome period within 5kb regions surrounding different promoter classes in ChIP-MNase datasets from DCs (blue) and fibroblasts (red). Promoters are grouped according to the expression levels of their associated transcripts, measured by RNA-seq and indicated in FPKM. Note that the mean nucleosomal period in fibroblasts is longer than in DCs when analysis is restricted to promoter regions, and also at all measured gene expression levels.
d. Example distribution of genomic distances between locations of nearby ChIP-MNase digestion fragment ends that are mapped on opposite strands. Only positive offsets are shown, representing reads that point towards each other. The distance of the major offset between fragment ends on opposite strands, which corresponds to the first peak in the distribution, reflects the major size of fragments that are protected from MNase digestion (the nucleosome 'footprint').
e. Mean nucleosomal footprint in replicate ChIP-MNase samples from DCs and fibroblasts. Note that unlike the nucleosomal period (panels a-c and figure 2a), which is determined entirely by the *in vivo* nucleosome position, the footprint may also be affected by sample preparation steps, which could contribute to greater variance between replicates. Dots indicate replicate samples; lines denote mean values.

f: Detection of TSS-proximal subnucleosomal fragments

Heatmap of occupancy levels surrounding active promoters in fibroblasts measured by H3K4me1 ChIP-MNase-seq analysis of 140-180bp (nucleosome footprint-sized) DNA fragments (left) or 50-100bp (subnucleosomal) DNA fragments (right); promoters are sorted by the predicted start site of antisense transcription (as[22]); sidebar indicates corresponding gene expression levels. As reported previously[36,92], TSS-proximal regions which exhibit depleted occupancy by nucleosome-sized particles nevertheless appear to be partially protected by digestion-sensitive (or subnucleosome-sized) particles; these have been proposed to reflect the presence of 'fragile nucleosomes'[30,93], but might also represent binding by other particles that differ from canonical nucleosomes[34-36].

Supplementary figure 5

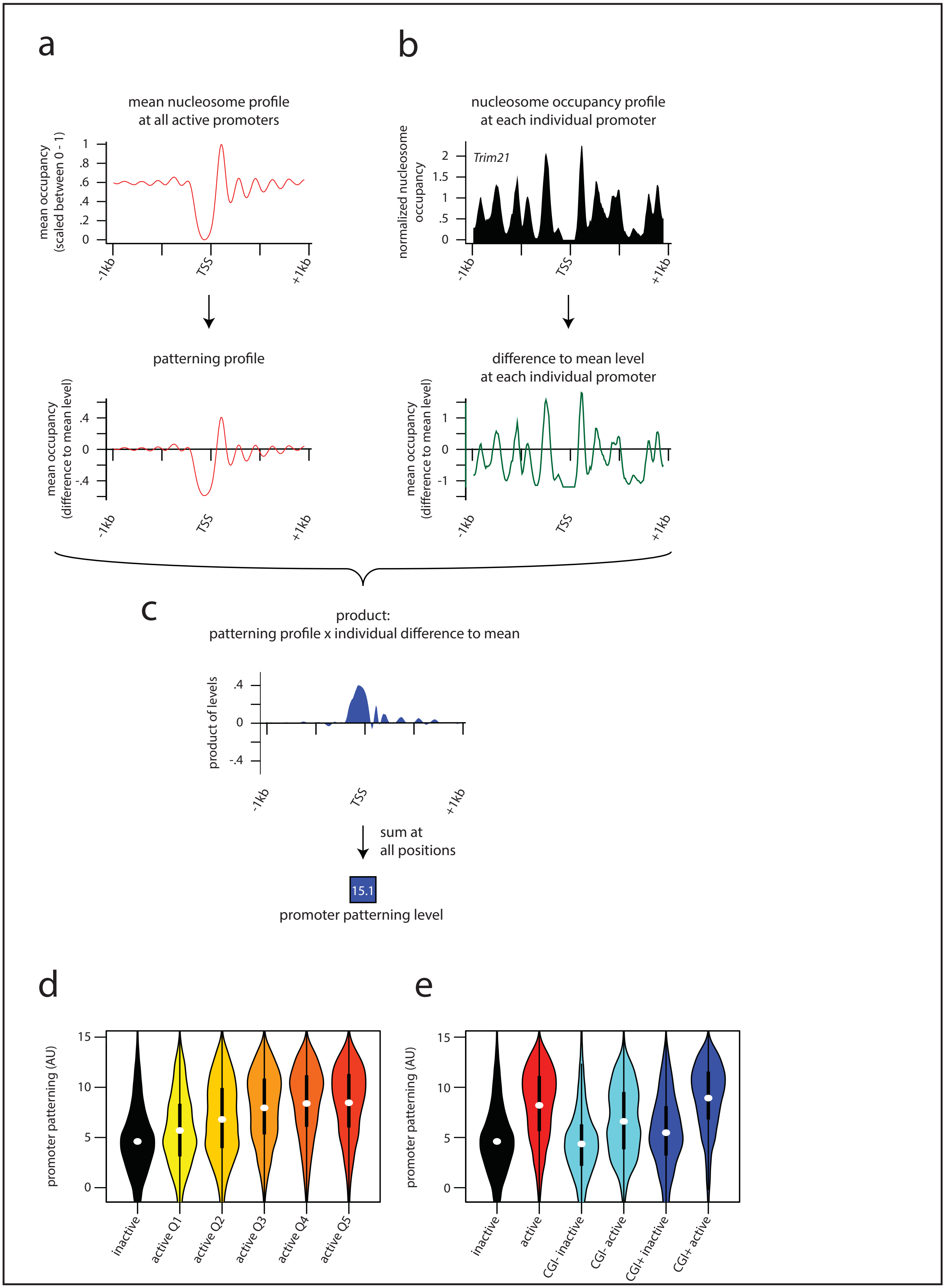

Supplementary figure 5

a-c. Quantitation of promoter patterning

a. The profile of mean nucleosome occupancy across all active promoters ±1kb was re-scaled to span the range from zero to one (representing the lowest and highest observed mean occupancy levels, respectively; upper panel). A patterning profile was generated by subtracting the mean occupancy level across the entire 2kb interval: thus, high-magnitude positive or negative values reflect the promoter positions with strongly favoured or disfavoured nucleosome occupancies at active promoters (lower panel).
b. Similarly, the normalized nucleosome occupancy profile across each individual promoter (upper panel; illustrated by the profile across the *Trim21* promoter) was transformed by subtracting the mean normalized nucleosome occupancy level across all promoters to generate a profile of differences to the mean level at each position (lower panel).
c. At each promoter, the value of the patterning profile (panel a) was multiplied by the value of the difference to the mean at that promoter (panel b) in every 10bp bin across the promoter region ±1kb (upper panel). This has the effect of heavily weighting nucleosome occupancies at highly favoured or disfavoured locations, which primarily encompass the NDR at the TSS, the +1 nucleosome, and gaps between the first few phased nucleosomes. The sum of the resulting products at all promoter regions was used to define the overall patterning level at each promoter.
Note that for hierarchical clustering of individual promoters, the same procedure was applied to the square-root of individual promoter normalized nucleosome occupancies, to reduce the disruptive effect of aberrantly high occupancy levels at isolated positions in a small fraction of promoters.

d,e: Quantified nucleosome patterning at different promoter classes

Promoter patterning levels were quantified as the closeness of the nucleosome occupancy profile at each promoter to the mean pattern at all active promoters in DCs; high values indicate highly 'patterned' promoters with a clear NDR, and typically-positioned +1 nucleosome and 'phased' downstream nucleosomes; low values indicate 'non-patterned' promoters which lack an NDR and/or at which nucleosome positions differ substantially from the typical distribution.
d. Patterning levels of promoters of non-expressed genes ('inactive'), and those of expressed genes divided into 5 equal quantiles of increasing activity level ('Q1' - 'Q5'). Note that all sets of active promoters contain 'patterned' promoters that significantly exceed the fraction among inactive promoters. Percentage of promoters with patterning greater than the highest quartile of inactive promoters: Q1: 38% ($p=5.5\times10^{-36}$); Q2: 50% ($p=8.2\times10^{-124}$); Q3: 63% ($p=1.2\times10^{-283}$); Q4: 70% ($p=4.9\times10^{-388}$); Q5: 70% ($p=3.9\times10^{-393}$).
e. Patterning levels of all inactive promoters; all active promoters; and inactive or active promoters separated according to whether their sequences correspond to a CpG island ('CGI+' and 'CGI-'). Percentage of promoters with patterning greater than the highest quartile of inactive promoters: active: 67% ($p=5.2\times10^{-1090}$); CGI- inactive: 22% ($p\approx1$); CGI- active: 48% ($p=3.7\times10^{-5}$); CGI+ inactive: 37% ($p=7.9\times10^{-13}$); CGI+ active: 76% ($p=5.7\times10^{-1084}$). All p-values are two-tailed Mann-Whitney U tests.

Supplementary figure 6

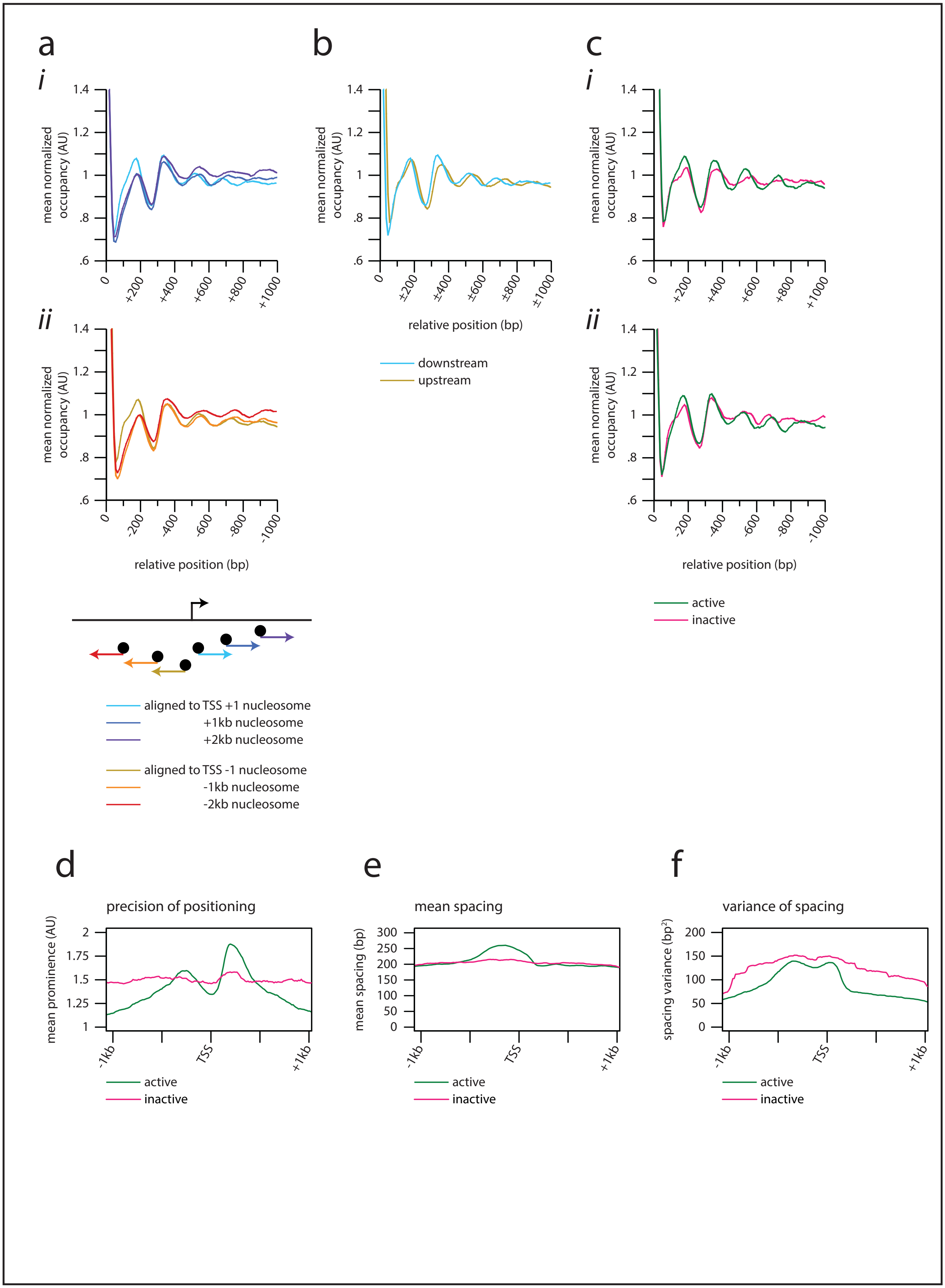

Supplementary figure 6

a-c: Nucleosome phasing at promoters

Nucleosome occupancy profiles across genomic intervals aligned to predicted nucleosome positions. In panels a-c, nucleosome occupancies at each individual locus are re-aligned to the position of the predicted nucleosome midpoint at or closest to the indicated position, and the occupancy profile represents the mean of all re-aligned loci. Thus, the very high value at position 0 (corresponding to the position to which each locus is aligned) is a consequence of the alignment strategy. Each profile is normalized to a mean value of 1 across the interval shown, to enable direct comparison of the different curves.
a. Nucleosome phasing at different distances downstream (i) or upstream (ii) of transcriptional start sites. Profiles are aligned to the predicted positions of +1 (cyan) or -1 (yellow) nucleosomes, or of the closest nuclesomes to locations at +1kb (blue), +2kb (purple), -1kb (orange) or -2kb (red) relative to each TSS. Although the nucleosome immediately adjacent to +1 and -1 nucleosomes show greater prominence than those adjacent to more distal nucleosomes, the subsequent magnitude of phasing is similar at all distances from the TSS. Note that the proximal downstream nucleosomes (in the range 0 to +1kb relative th the TSS) show a detectably shorter period.
b. Nucleosome phasing upstream and downstream of TSSs. Profiles are aligned to the predicted positions of +1 (downstream, cyan) or -1 (upstream, yellow) nucleosomes. The magnitude of phasing is similar for upstream and downstream nucleosomes, although downstream nucleosomes show a shorter period.
c. Nucleosome phasing at active (green) and inactive (red) promoters, downstream (i) or upstream (ii) of TSSs. Profiles are aligned to the predicted positions of +1 (downstream, i) or -1 (upstream, ii) nucleosomes. The magnitude of phasing is greater at active than inactive promoters, most obviously for downstream nucleosomes (located within the transcribed region).

d-f: Precision of nucleoeoms positioning and mean & variance of internucleosome spacing at promoters

Profiles of mean prominence of peaks (d), or of the mean (e) and variance (f) of peak-peak spacing, at active (green) and inactive (magenta) promoters. Observed nucleosome phasing - the periodicity of profiles of mean occupancies averaged across multiple genomic loci - depends on precise nucleosome placement in the same position at all alleles of each individual locus (quantified by prominence, d); as well as on consistent internucleosomal spacing at each locus (quantified by mean and variance, e,f). Inactive promoters display more-precise nucleosome positioning at TSS-distal regions (higher mean prominences, d) but larger variation in internucleosome spacing (f), which together account for the modest reduction in observed phasing (panel c).

Supplementary figure 7

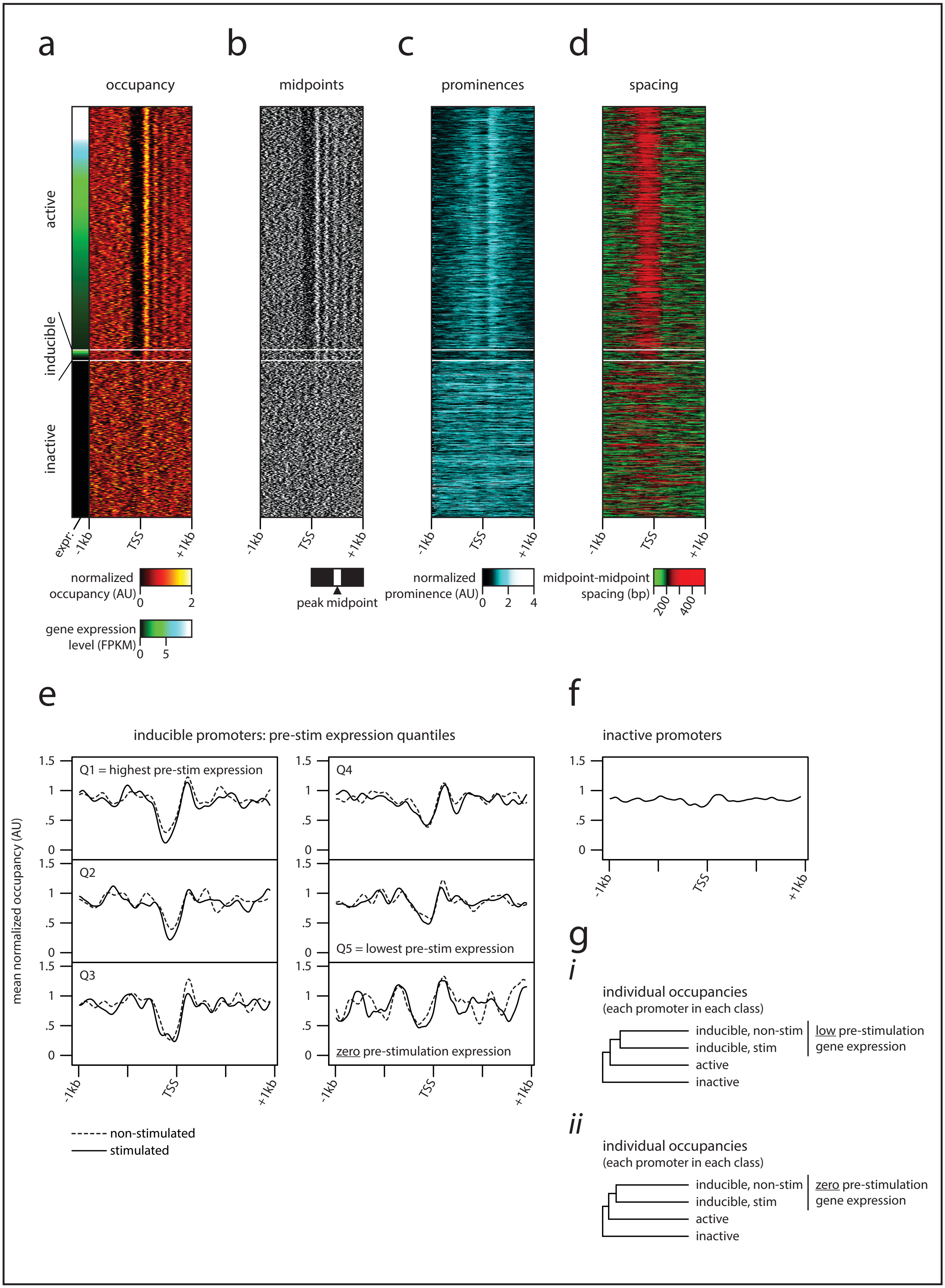

Supplementary figure 7

a-d: Nucleosome occupancies, predicted midpoints, peak prominences and internucleosome spacings at promoters.

Heatmaps of nucleosome occupancy levels (a), positions of predicted nucleosome midpoints (b; see figure 1e and supplementary figure 3c), peak prominences (c; reflecting the precision of nucleosome positioning at each allele; see supplementary figure 3c), and internucleosome spacing (d; calculated as the distance betweeen adjacent predicted midpoints) surrounding active (top), inducible (middle) and inactive (bottom) promoters in DCs; sidebar indicates corresponding gene expression levels (level for inducible promoters represents non-stimulated cells.

e-g: Nucleosome patterning at inducible promoters resembles that at active promoters

e,f. Profiles of mean nucleosomal occupancies at inducible (e) and inactive (f) promoters in non-stimulated (dotted lines) and LPS-stimulated (solid lines) DCs. Inducible promoters are divided into those with detectable pre-stimulation activity, grouped into 5 equal quantiles of increasing activity level according to pre-stimulation mRNA levels of the associated transcripts (Q1: highest - Q5: lowest), or those with no detectable transcripts in non-stimulated cells. Mean occupancy profiles of all inducible promoter groups display a clear NDR as well as a prominent +1 nucleosome.
g. Hierarchical clustering of nucleosome occupances from distinct sets of promoters, applied to individual levels at each promoter separately: i. including only inducible promoters with low pre-induction gene expression levels (so that pre-induction mRNA abundances associated with active promoters & inducible promoters do not overlap); ii. including only inducible promoters with no detectable transcripts in non-stimulated cells (so that the pre-induction absence of mRNA is identical between inactive & inducible promoters).

Supplementary figure 8

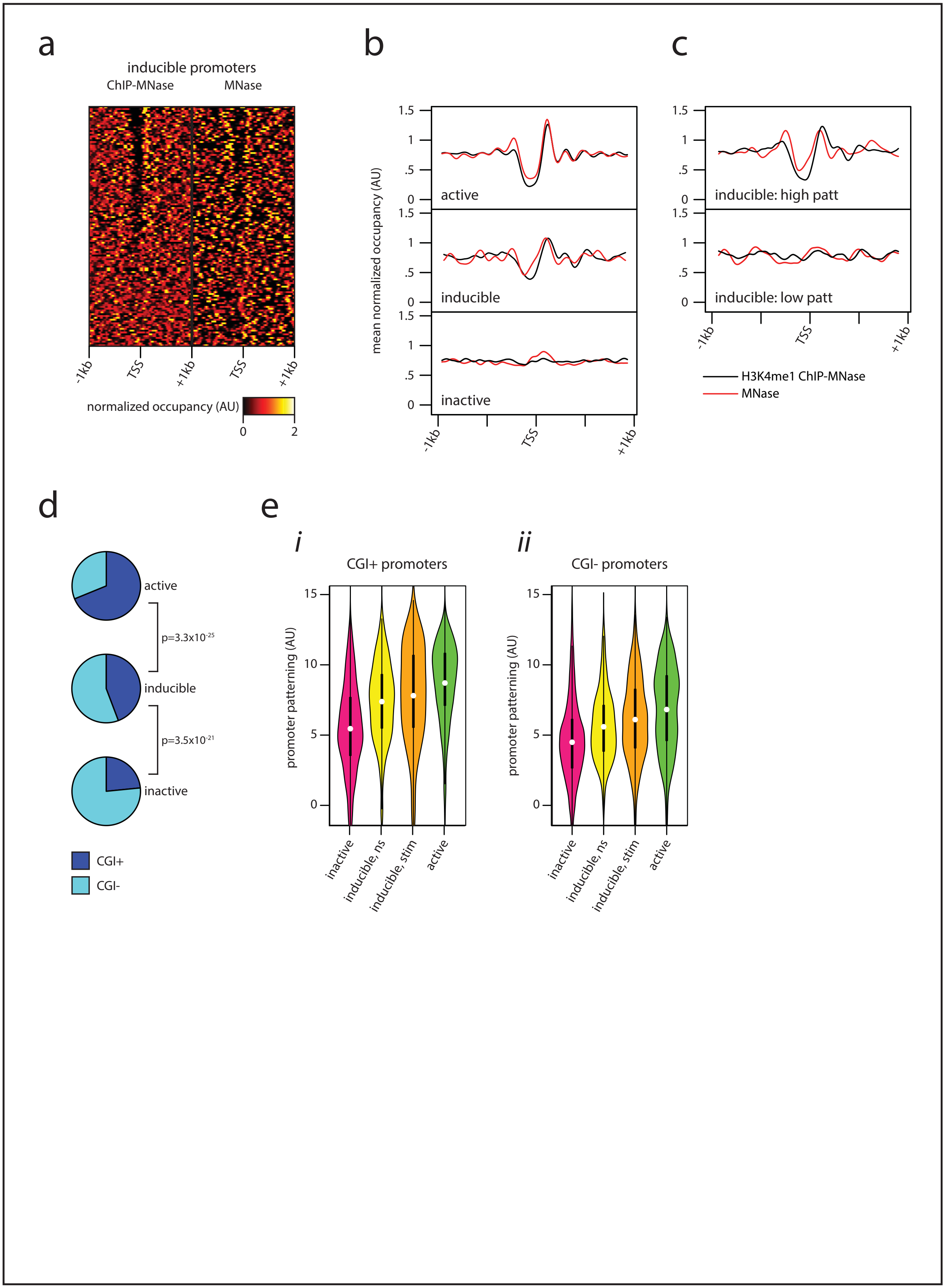

Supplementary figure 8

a-c: Nucleosome patterning at indudible promoters is detectable in whole-genome MNase-seq data

H3K4me1 ChIP-MNase profiles compared to genome-wide MNase profiles generated in the same cell-type (fibroblasts), to verify that patterning detected at inducible promoters does not arise as a consequence of preferential recovery of a minority of patterned alleles by the H3K4me1 ChIP-MNase technique.
a. Heatmap of nucleosome occupancy levels surrounding inducible promoters in non-stimulated fibroblasts, measured by H3K4me1 ChIP-MNase-seq, or by whole-genome MNase-seq, sorted by the the closeness of nucleosomal patterning at each promoter to the mean pattern at active promoters to reveal the heterogeneous patterning of inducible promoters.
b,c. Profiles of mean nucleosomal occupancies at inducible, active and inactive promoters (b), or at inducible promoters separated into subsets exhibiting high or low patterning (c), in non-stimulated fibroblasts, measured by H3K4me1 ChIP-MNase-seq, or by whole-genome MNase-seq[91]. Note that the level of coverage mapping to promoters in the whole-genome MNase-seq data analysed (27 reads kb^{-1}) is lower than the level attained by ChIP-MNase (≥50 reads kb^{-1} at all promoters analysed). Mean occupancy profiles of inducible promoter groups display a clear NDR as well as a prominent +1 nucleosome in both ChIP-MNase and MNase datasets (b), and inducible promoters that are assigned as high- or low-patterned based on ChIP-MNase retain these characteristics in MNase datasets (c; see also panel a).

d,e: Nucleosome patterning at CGI+ and CGI- promoters

d. Proportions of CGI+ (blue) and CGI- (cyan) promoters among active (top), inducible (middle) and inactive promoters.
e. Patterning levels of promoters of different classes of CGI+ (i) and CGI- (ii) promoters. Among both CGI+ and CGI- promoters, inducible promoters contain a significant number with high patterning levels compared to inactive promoters. Percentage of promoters with patterning greater than the highest quartile of all inactive promoters: CGI+ inducible, non-stimulated: 61% ($p=3.8 \times 10^{-26}$); CGI+ inducible, stimulated: 65% ($p=1.8 \times 10^{-30}$); CGI- inducible, non-stimulated: 34% ($p=1.3 \times 10^{-3}$); CGI- inducible, stimulated: 43% ($p=6.0 \times 10^{-10}$). All p-values are two-tailed Mann-Whitney U tests.

Supplementary figure 9

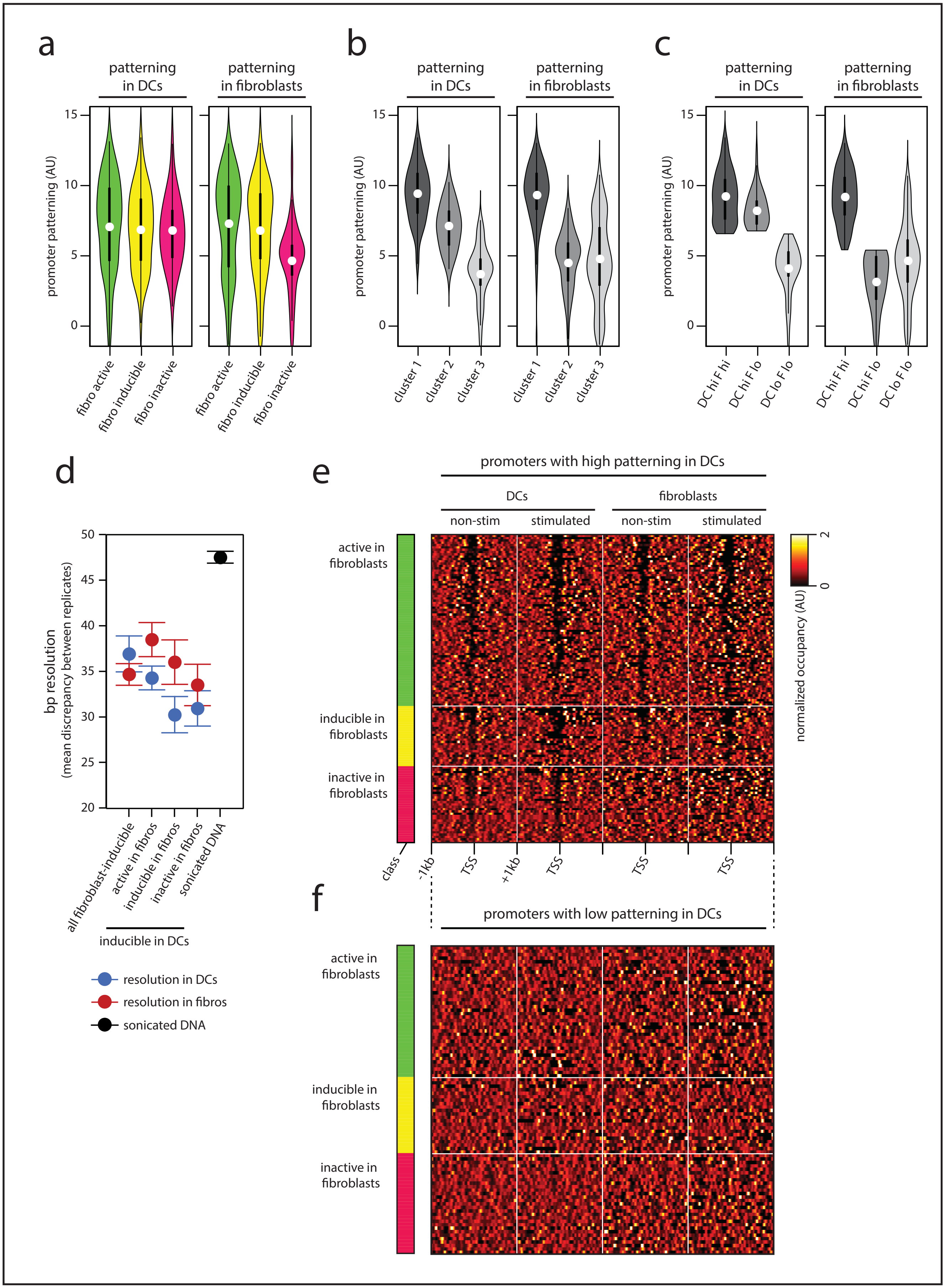

Supplementary figure 9

a-c: Quantified patterning levels of inducible promoter subsets.

a-c. Violin plots of quantified promoter patterning levels in DCs (left panels) and fibroblasts (right panels), at promoters that are inducible in DCs, grouped by their acvity class in fibroblasts (a, as figure 4a), sorted by hierarchical clustering of weighted nucleosome profiles (b, as figure 4b), or grouped by promoter nucleosome patterning levels (c, as figure 4c). (a) Differences between all groups in DCs, and between active and inducible promoters in fibroblasts, are non-significant ($p>0.16$ for all pairwise comparisons); significances of differences to inactive promoters in fibroblasts: active promoters: $p=2.5\times10^{-6}$; inducible promoters: $p=3.7\times10^{-4}$. (b) Significances of differences to cluster 3 in DCs: cluster 1: $p=4.8\times10^{-25}$; cluster 2: $p=3.7\times10^{-17}$; significances of differences to cluster 1 in fibroblasts: cluster 2: $p=4.2\times10^{-21}$; cluster 3: $p=1.0\times10^{-16}$; clusters 2 and 3 in fibroblasts are not significantly different ($p=0.84$). (c) Significances of difference between 'DC hi F hi' and 'DC lo F lo' groups in fibroblasts: $p=1.6\times10^{-17}$ (two-tailed Mann-Whitney U test). Thick bars indicate limits of quartiles; dots indicate means.

d: Resolution of predicted nucleosome positions, at distinct promoter sets.

Resolution of predicted nucleosome positions mapping to distinct promoter sets, calculated as the mean discrepancy between replicate samples (as figure 1g). Note that among DC-inducible promoters, the accuracies of predicted nucleosome positions at promoters that are inactive in fibroblasts is comparable or higher than those of other promoter classes, indicating that the absence of detectable 'patterning' at these promoters does not arise as a consequence of low data quality or resolution. Error bars indicate standard error of the mean (SEM). Source data are provided as a source data file.

e,f. The activities in fibroblasts of promoters that are inducible & patterned in DCs can be predicted from their nucleosome patterning

Heatmap of nucleosome occupancy levels surrounding promoters that are inducible in DCs, in non-stimulated & LPS-stimulated DCs (left panels) and in non-stimulated and TNF-α-stimulated fibroblasts (right panels), as in figure 4a. Promoters are grouped by their acvity class in fibroblasts, indicated by sidebar: active (top, green), inducible (middle, yellow), or inactive (bottom, red), and sorted within this by their level of patterning.
e. Promoters with high patterning in DCs: note that promoters that are active or inducible in fibroblasts exhibit predominantly high patterning, with a prominent NDR and often a typically-positioned +1 nucleosome; promoters that are inactive in fibroblasts do not exhibit any clear promoter nucleosome patterning. Thus, for this set of promoters, the level of nucleosome patterning in fibroblasts is partially predictive of their activity in this cell-type.
f. Promoters with low patterning in DCs: note that promoters within this set do not exhibit any clear promoter nucleosome patterning, irrespectively of whether they are transcriptionally active, inducible or inactive in this cell-type.

Supplementary figure 10

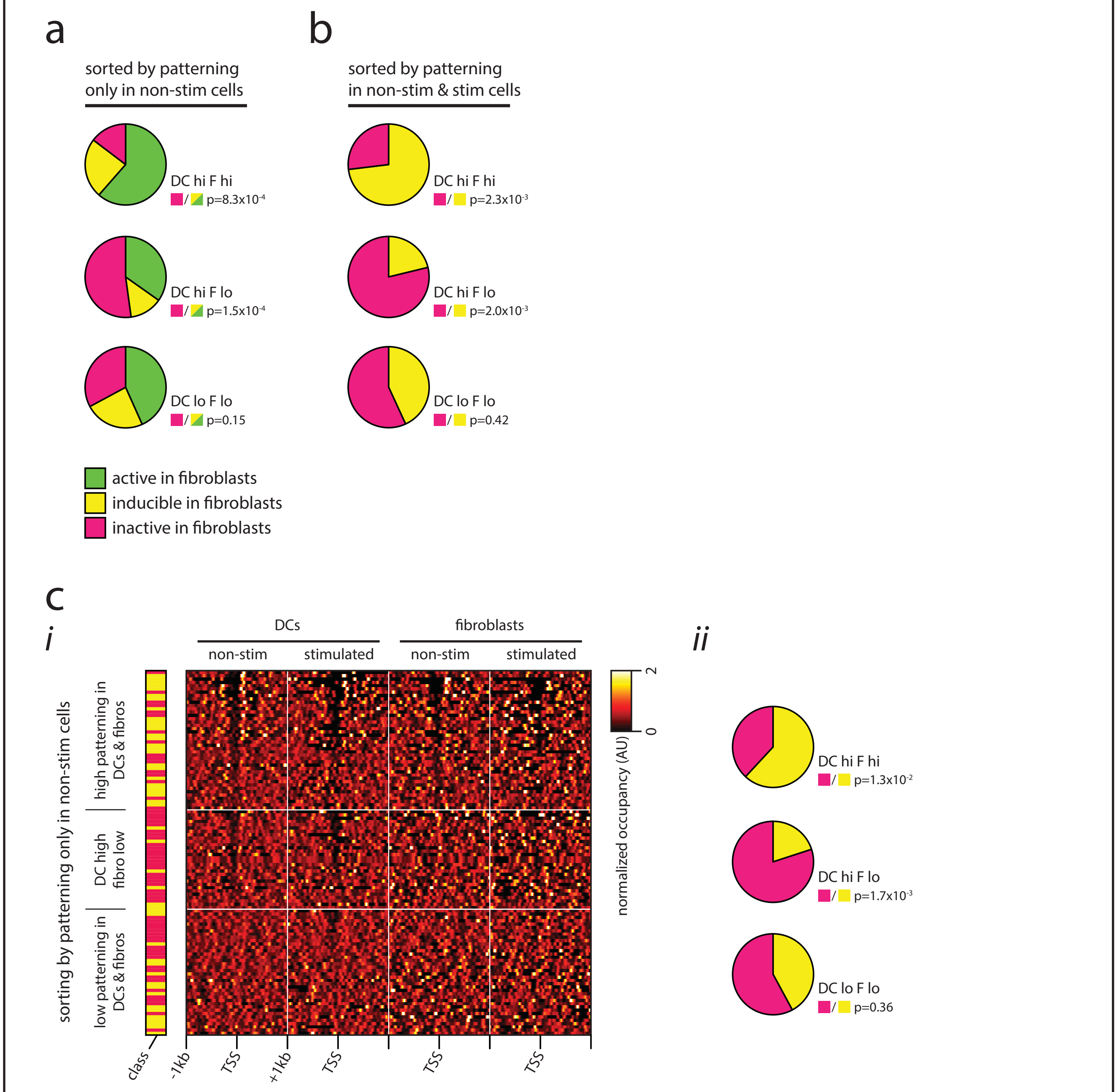

Supplementary figure 10

a-c: Nucleosome patterning in non-stimulated cells can predict the stimulus-inducible behaviour of many promoters

a. Pie charts summarizing the behaviour in fibroblasts of promoters that are inducible in DCs, as in figure 4c, here grouped by promoter nucleosome patterning levels only in non-stimulated cells. P-values indicate the significance of enrichment or depletion of promoters that are inactive compared to those that are active or inducible in fibroblasts. Active and inducible promoters were not statistically distinguishable ($p>0.05$ for all groups).
b. Pie charts summarizing the behaviour in fibroblasts of promoters that are inducible in DCs, grouped as in figure 4c, here considering only promoter classes that are not active in non-stimulated fibroblasts.
c. i. Heatmap of nucleosome occupancy levels as in figure 4a-c, here grouped by promoter nucleosome patterning levels only in non-stimulated cells and considering only promoter classes that are not active in non-stimulated fibroblasts. ii. Pie charts summarizing the proportion of each promoter class in each group. P-values indicate the significance of enrichment or depletion of promoters that are inactive compared to those that are inducible in fibroblasts. Among the groups of promoters that exhibit high patterning in DCs, 82% of fibroblast-inducible promoters exhibit high patterning in non-stimulated fibroblasts (expected: 58%; $p=1.6x10^{-3}$), and 60% of fibroblast-inactive promoters exhibit low patterning in non-stimulated fibroblasts (expected: 42%; $p=6.3x10^{-3}$). All p-values are two-tailed binomial tests.

Supplementary figure 11

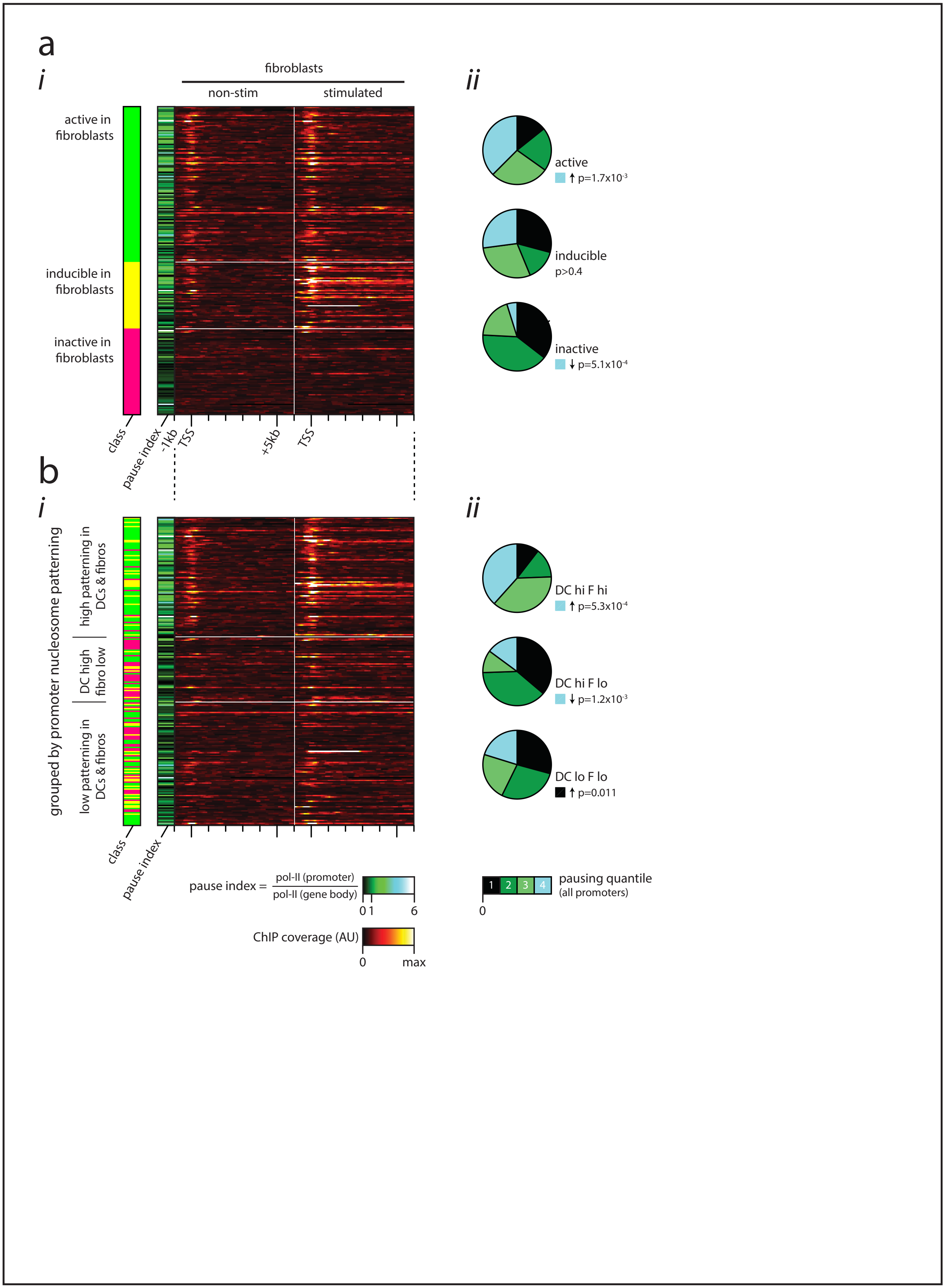

Supplementary figure 11

a,b: Promoters that are consistently active or inducible in different cell-types are enriched for paused RNA pol-II

a. i. Heatmaps of ChIP coverage of RNA pol-II S7P surrounding promoters that are inducible in DCs, grouped by their activity class in fibroblasts as in figure 4a and indicated by left sidebar. The ‘Pause index’ sidebar indicates the ratio of pol-II S7P density in non-stimulated cells at each promoter (±500bp) to the mean density across the first 5kb of the gene body. ii. Pie charts summarizing the proportion of promoters in each group that have pause indices falling within each of 4 quantiles, based on the distribution of pause indices of all annotated promoters. P-values indicate the significance of enrichment or depletion of promoters with the indicated pausing indices compared to the fraction in all promoters (two-tailed binomial test).
b. i. Heatmaps of ChIP coverage of RNA pol-II S7P as in panel a, grouped by promoter nucleosome patterning levels as in figure 4c. ii. Pie charts summarizing the proportion of promoters in each group with pausing indices as in panel j. P-values indicate the significance of enrichment or depletion of promoters with the indicated pausing indices compared to the fraction in all promoters (two-tailed binomial test). Paused promoters are strongly enriched within the group of promoters that are patterned in fibroblasts. Note that the distribution of pause indices at all fibroblast-inducible promoters is not significantly different from the distribution at all promoters. Similar results were obtained analysing promoter occupancy by total RNA pol-II (based on ChIP data using an antibody specific for the Rpb N-terminus; not shown).

Supplementary figure 12

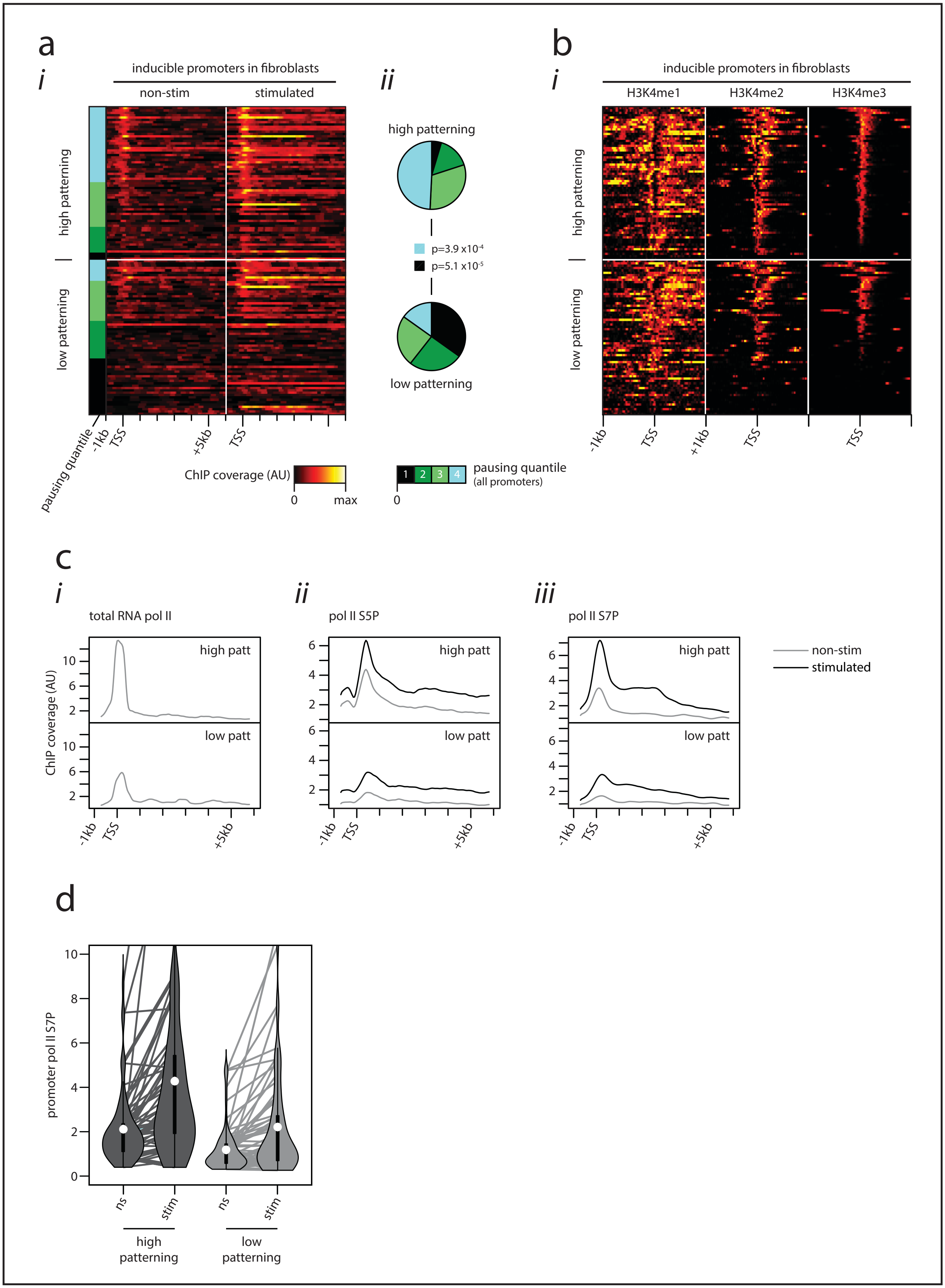

min stroke = 0.35pt

min text size (not specified) = 6pt

Supplementary figure 12

a-d: Inducible promoters with highly-patterned nucleosomes are enriched for paused RNA pol-II and H3K4 di- and trimethylation

a. i. Heatmaps of ChIP coverage of RNA pol-II S7P surrounding all promoters that are inducible in fibroblasts, grouped by nucleosome patterning levels in non-stimulated cells. ii. Pie charts summarizing the proportion of promoters in each group with pausing indices as in panel j. P-values indicate the significance of enrichment or depletion of promoters with the indicated pausing indices (two-tailed binomial test). Paused promoters are enriched within the patterned group of promoters.
b. Heatmaps of ChIP coverage of H3K4me1 (left), H3K4me2 (middle) & H3K4me3 (right) surrounding all promoters that are inducible in fibroblasts as in supplementary figure 2a, grouped by nucleosome patterning levels in non-stimulated cells as in panel a. Note that the majority of patterned inducible promoters, but only a subset of non-patterned promoters, are positive for H3K4me3 and H3K4me2.
c. i. Profiles of mean ChIP signals for total RNA pol II (i), RNA pol II S5P (ii) and RNA pol II S7P (iii) across promoters with high patterning (top) or low patterning (below) in non-stimulated fibroblasts. Grey lines: no stimulation; solid lines: TNF-α-stimulation.
d. Violin plots of quantified mean promoter ChIP signal (TSS ±500bp) for RNA pol II S7P all promoters that are inducible in fibroblasts, grouped by nucleosome patterning levels in non-stimulated cells as in panel a. The level of the signal at each promoter in non-stimulated (ns) cells is joined to the level at the same promoter in stimulated (stim) cells, to indicate the behaviour of individual promoters. Thick bars indicate limits of quartiles; dots indicate means.

Supplementary figure 13

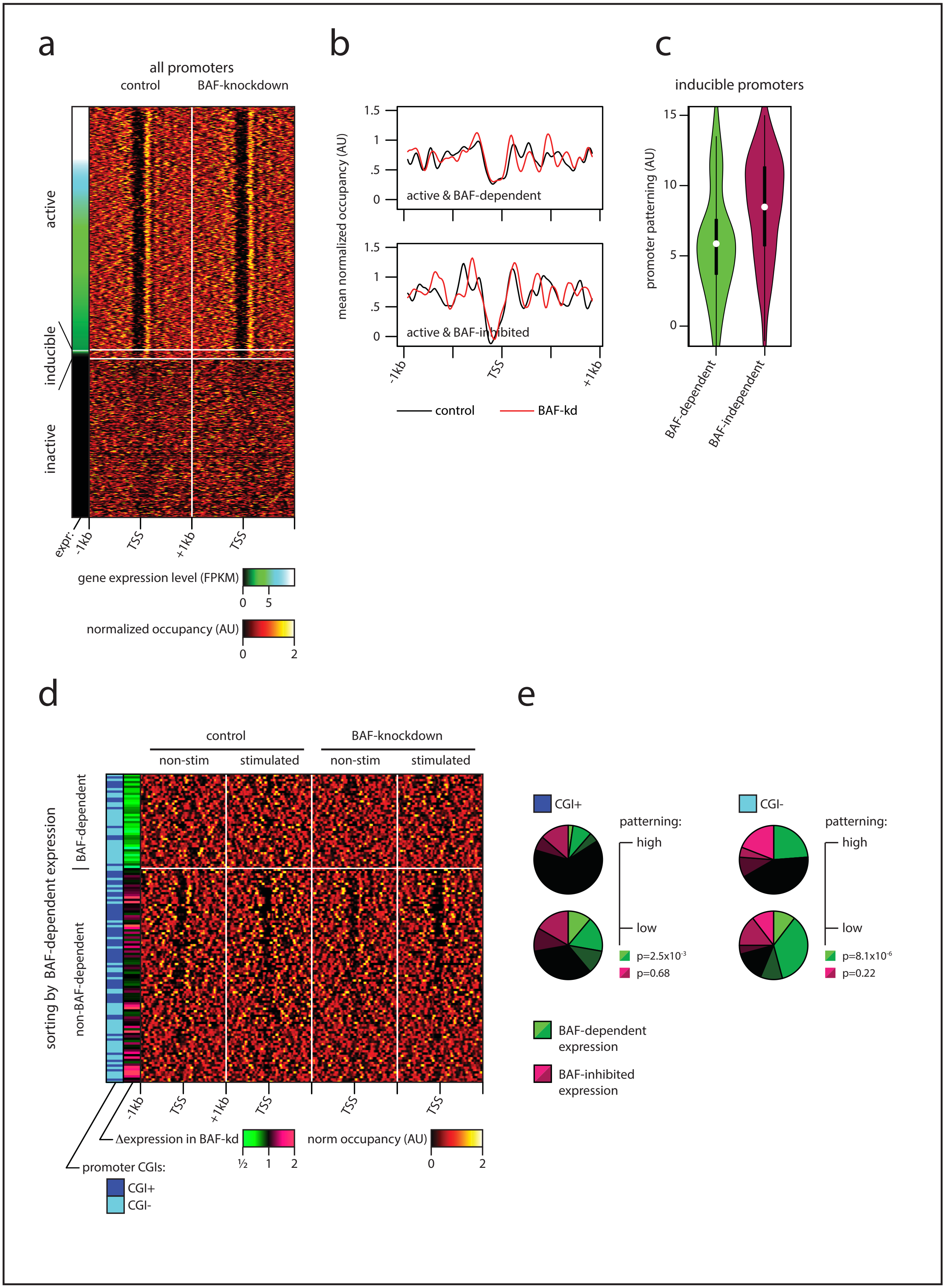

Supplementary figure 13

a,b: Most promoters do not display clearly disrupted nucleosome positioning in BAF-knockdown cells

a. Heatmap of nucleosome occupancy levels surrounding active (top), inducible (middle) and inactive (ii) promoters in fibroblasts, in control and BAF-knockdown cells; sidebar indicates corresponding gene expression levels in unstimulated, normal cells.
b. Profiles of mean nucleosomal occupancies at the subset of active promoters that display BAF-dependent (top) or BAF-inhibited (bottom) activity in fibroblasts, measured by ChIP MNase in control (black) or BAF-knockdown (red) fibroblasts.

c,d: Inducible promoters with BAF-dependent activity are enriched for low patterning levels.

c. Violin plots of quantified promoter patterning levels at inducible promoters in fibroblasts, that are BAF-dependent (green, left) or non-BAF-dependent (red, maroon). Significance of difference in patterning between BAF-dependent and non-BAF-dependent inducible promoters: $p=3.4x10^{-4}$ (two-tailed Mann-Whitney U test). Thick bars indicate limits of quartiles; dots indicate means.
d. Heatmap of nucleosome occupancy levels surrounding inducible promoters in control (left panels) and in BAF-knockdown (right panels) fibroblasts, grouped into BAF-dependent and non-BAF-dependent sets of promoters. These are the same data as shown in panel d of figure 5, here reordered by BAF-dependence. Sidebars indicate presence or absence of promoter CGIs (outer sidebar) or the level that inducible gene expression from each promoter is BAF-dependent or -inhibitied (inner sidebar).

e. Both CGI+ and CGI- promoters with high patterning are depleted for BAF-dependent activity

Pie charts summarizing the proportion of genes with BAF-dependent (green) or BAF-inhibited (red) expression, among CGI+ (left) and CGI- (right) promoters with high (top) or low (bottom) levels of nucleosome patterning. P-values indicate the significance of enrichment within low-patterned promoters, for CGI+ and CGI- groups.

Supplementary figure 14

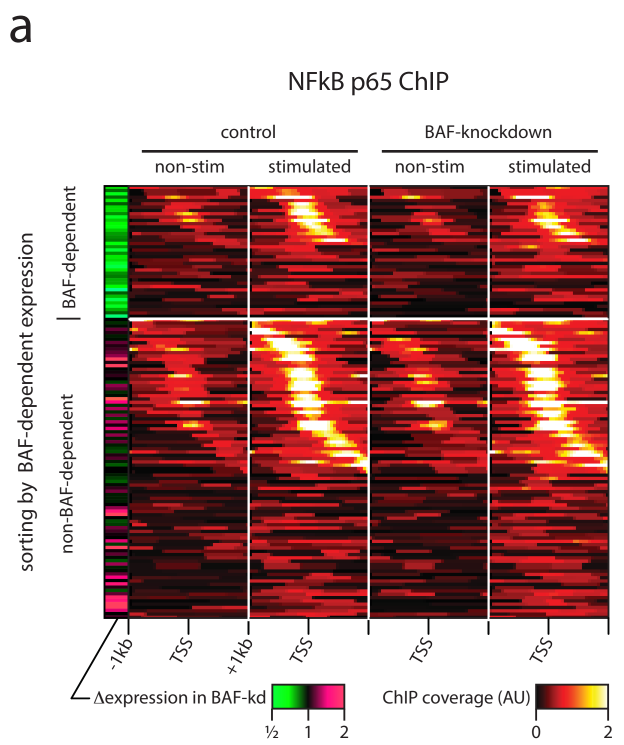

Supplementary figure 14

a. NFkB p65 recruitment is not impaired at BAF-dependent inducible promoters

Heatmaps of ChIP coverage of NFkB p65 surrounding promoters that are inducible by TNF-α in fibroblasts, in non-stimulated & TNF-a-stimulated conrol fibroblasts (left panels) and in BAF-knockdown fibroblasts (right panels). Promoters are grouped into BAF-dependent and non-BAF-dependent sets, and the sidebar indicates the level that inducible gene expression from each promoter is BAF-dependent or -inhibitied (as in supplementary figure 13d). NFkB p65 is a major effector of the TNF-α response in fibroblasts, and around half of responsive genes exhibit inducible p65 recruitment to their promoter regions, as well as other genes that recruit p65 to more distal sites (not displayed here). The set of promoters that recruit p65, as well as the level of p65 recruitment, is not significantly impaired by BAF-knockdown.

Supplementary figure 15

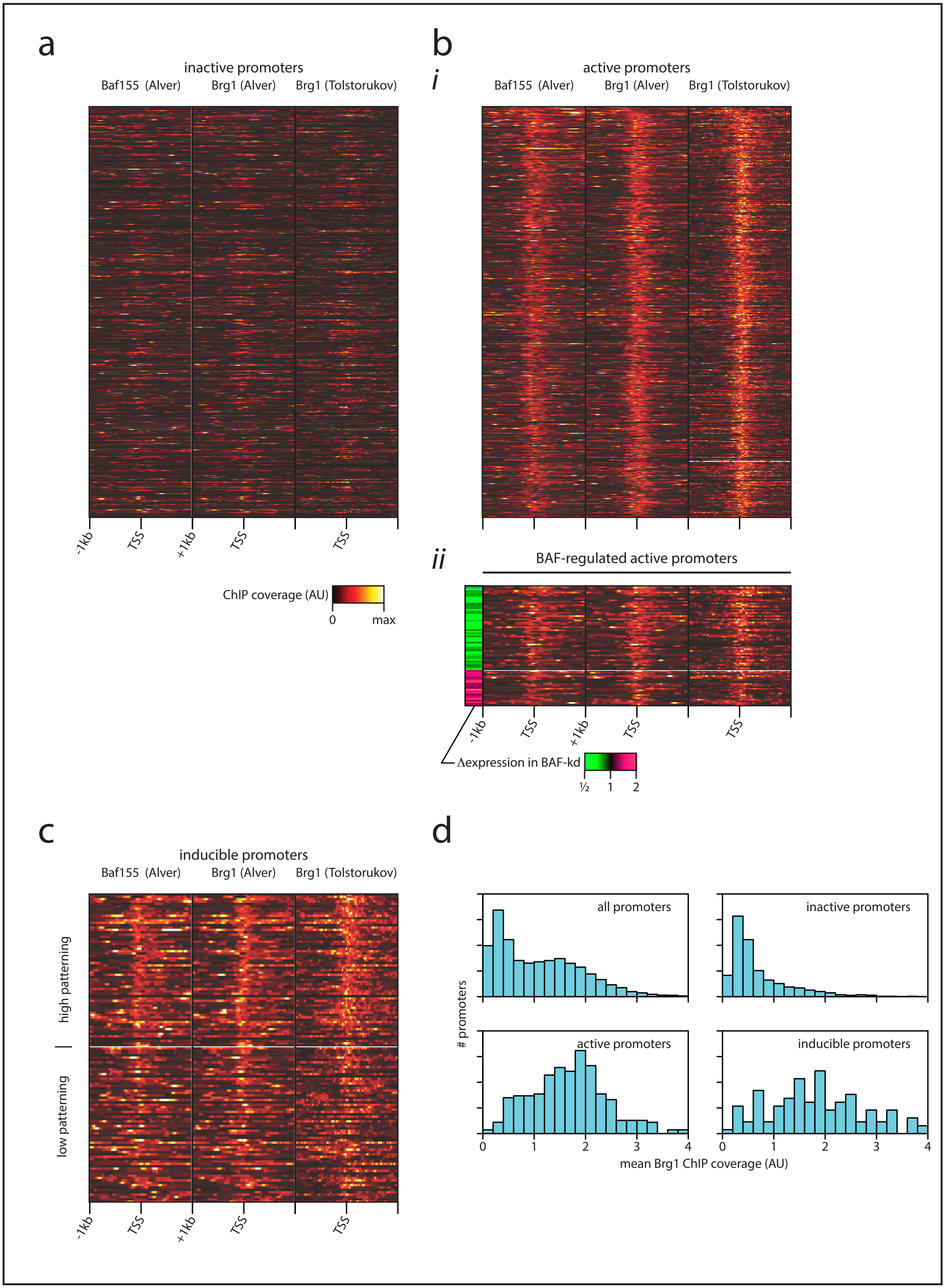

Supplementary figure 15

a-d: The BAF complex binds strongly to all inducible promoters in fibroblasts

a-d. Heatmaps of ChIP coverage of Baf155 (left;[61] & Brg1 (middle;[61] and right;[62] surrounding promoters that are inactive (a), active (b, panel i) or inducible (c) in fibroblasts. Panel b ii shows the subset of active promoters that display BAF-dependent (top) or BAF-inhibited (bottom) activity in fibroblasts.
d. Histograms of the mean levels of Brg1 ChIP[62] coverage at all promoters (top left), inactive promoters (top right), active promoters (bottom left) and inducible promoters (bottom right) in fibroblasts, showing that the majority of all active and inducible promoters are bound by this component of the BAF complex.

Supplementary figure 16

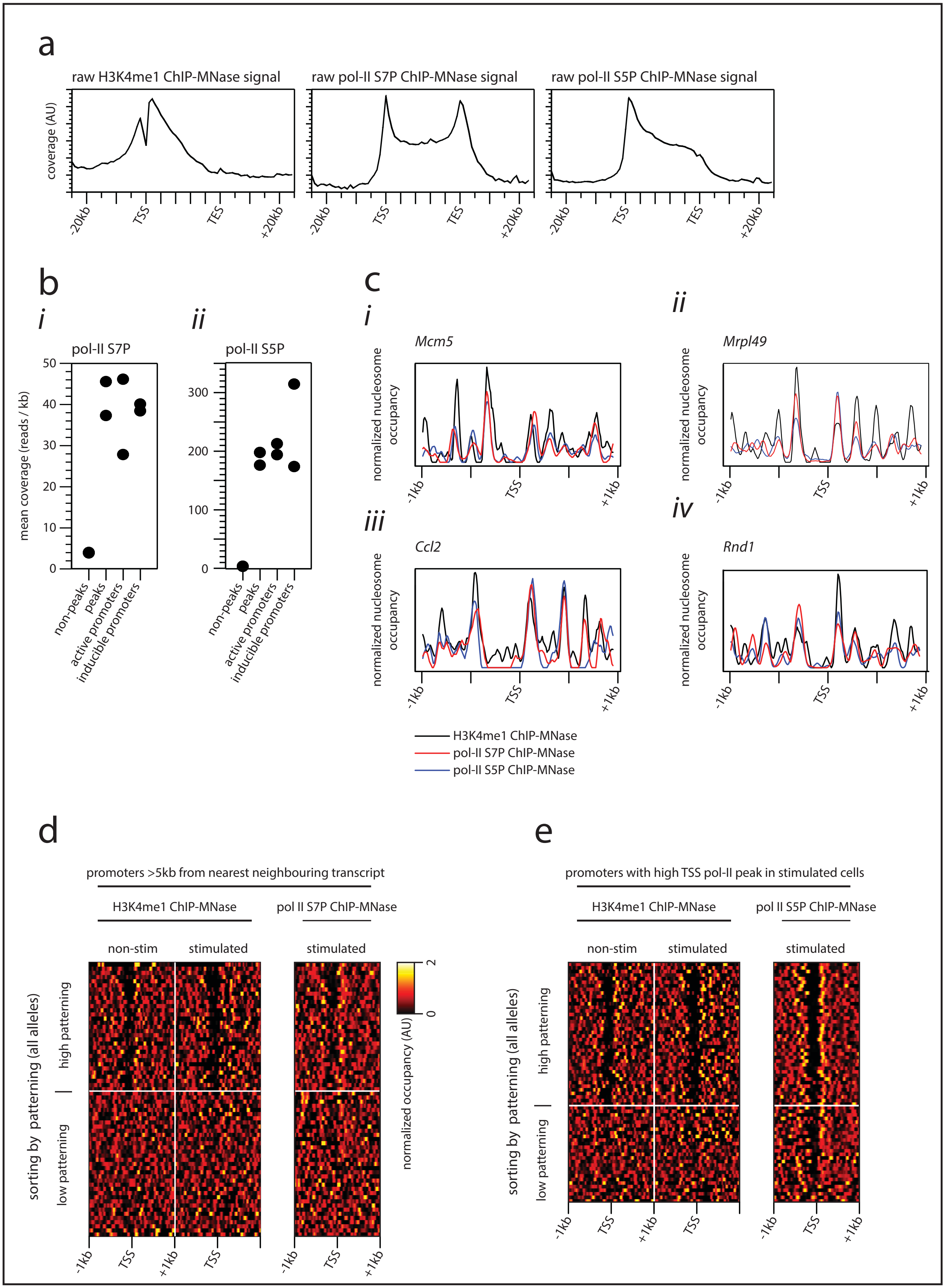

Supplementary figure 16

a,b: Enrichment levels of RNA pol-II S7P and S5P ChIP MNase

a. Profiles of mean, non-normalized, ChIP MNase signals across gene transcribed regions (scaled proportionally to gene length, so that all transcribed regions occupy the same display interval) and across 25kb upstream and downstream genomic regions. Left: H3K4me1 ChIP MNase; Middle: RNA pol-II S7P ChIP MNase; Right RNA pol-II S5P ChIP MNase. The signal for pol-II S7P spans the entire transcribed interval at high coverage, whereas the signal for pol-II S5P is enriched at the TSS and regions corresponding to early stages of transcription, and diminishes along the transcribed region.
b. Specificity of pol-II S7P and S5P ChIP, measured by coverage levels at ChIP peak regions (defined as the genomic intervals with the 5% highest coverage, excluding the top 0.5% [that may contain possible alignment or genome assembly artefacts]), non-peak regions (defined as all genomic intervals with less than median coverage), and at 2kb intervals surrounding active or inducible promoters. pol-II S7P ChIP achieves a target enrichment (peak ÷ non-peak coverage) of around 12-fold, indicating that even promoters that are active in only 8% of cells (or at 8% of alleles) can be effectively separated and analysed in this way. Likewise, pol-II S7P ChIP achieves a target enrichment of around 40-fold.

c: Comparison of H3K4me1 ChIP-MNase, pol-II S7P ChIP-MNase and pol-II S5P ChIP-MNase at example promoters

Normalized nucleosome occupancy profiles in non-stimulated fibroblasts across individual promoters of the *Mcm5* (i; expressed in fibroblasts), *Mrpl49* (ii; expressed in fibroblasts), *Ccl2* (iii; inducible in fibroblasts) and *Rnd1* (iv; inducible in fibroblasts) genes, measured by H3K4me1 ChIP-MNase-seq (black), RNA pol-II S7P ChIP-MNase-seq (red) or RNA pol-II S5P ChIP-MNase-seq (blue).

d,e: pol-II ChIP-MNase at selected subsets of inducible promoters

d. Heatmaps of nucleosome occupancy levels assayed by H3K4me1 ChIP-MNase (left) or RNA pol-II S7P ChIP-MNase (right), surrounding promoters that are inducible in fibroblasts and that are further than 5kb from the nearest neighbouring transcript, to minimize the possibility that any non-transcribing promoter alleles could be inadvertently recovered due to their presence on large chromatin fragments that are immunoprecipitated through pol-II transcribing an adjacent gene. Promoters are grouped by nucleosome patterning levels in non-stimulated cells.

e. Heatmaps of nucleosome occupancy levels assayed by H3K4me1 ChIP-MNase (left) or RNA pol-II S5P ChIP-MNase (right), surrounding promoters that are inducible in fibroblasts and that exhibit a high peak of pol-II at the TSS in stimulated cells (defined as pol-II at TSS ±500bp at least twice the level detected in the region -1kb to -500bp; see figure S4g), to enrich for promoters with a higher fraction of initiating alleles (at which a pol-II S5P ChIP-MNase signal is more-likely to arise from initiating, rather than early-elongating, pol-II).

Supplementary figure 17

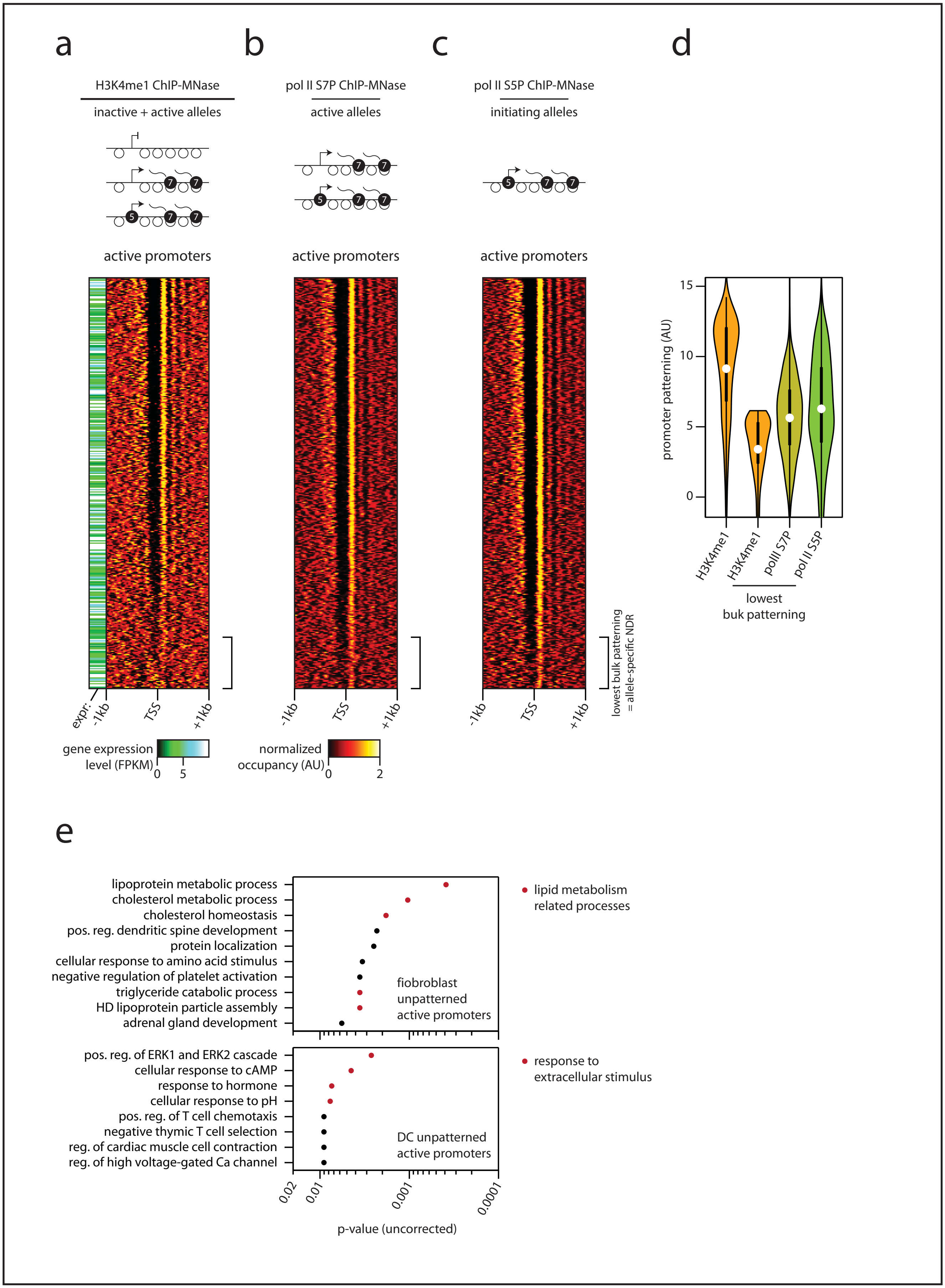

Supplementary figure 17

a-d: Allele-specific patterning at a subset of active promoters

a-c. Heatmaps of nucleosome occupancy levels surrounding promoters that are active in fibroblasts, assayed by H3K4me1 ChIP-MNase to detect both active and inactive promoter alleles (a), by RNA polII S7P ChIP-MNase to detect only active alleles (b), or by RNA polII S5P ChIP-MNase to enrich for alleles undergoing transcription initiation (c). Promoters are sorted by the level of patterning quantified by H3K4me1 ChIP-MNase; sidebar indicates corresponding gene expression levels. Promoters that exhibit the lowest levels of patterning and no NDR at all alleles, but at which increasing levels of patterning are detectable at polII S7P-marked and polII S5P-marked alleles are indicated ('allele-specific NDR').
d. Violin plots of quantified promoter patterning levels at active promoters in fibroblasts, at all alleles (orange; H3K4me1 ChIP-MNase) of all promoters (left violin) or of those promoters that exhbit the lowest levels of patterning (centre left violin), or at only active promoter alleles (olive-coloured; polII S7P; centre right violin), or only at initiating promoter alleles (green; polII S5P; right violin). Among active promoters with the lowest levels of bulk patterning at all alleles, 45% display higher patterning levels at active alleles and 51% display higher patterning levels at initiating alleles. Significance of difference in patterning levels compared to those quantified by H3K4me1 ChIP MNase: polII S7P ChIP-MNase: $p=1.7x10^{-45}$; polII S5P ChIP-MNase: $p=4.4x10^{-53}$ (two-tailed Mann-Whitney U test). Thick bars indicate limits of quartiles; dots indicate means.

e: Enrichment for annotated biological pathways among active genes with promoters that exhiibit low patterning levels

P-values corresponding to the significance of enrichments of the most highly-enriched pathways among low-patterned active promoters in fibroblasts (upper panel) or in DCs (lower panel). P-values represent two-tailed binomial tests comparing low-patterned active promoters to all active promoters in each cell type, without correction for multiple testing. Biological pathways related to lipid metabolism (consistent with developmental regulation due to the pre-adipocyte-like status of 3T3 fibroblasts) or responses to extracellular stimuli (consistent with stimulus-inducible genes in DCs) are highlighted in red.
