# Supplementary material for: Role of cell-type specific nucleosome positioning in inducible activation of mammalian promoters
Source: Nat Commun. 2020 Feb 26;11:1075. doi: 10.1038/s41467-020-14950-5 (PMC7044431; doi:10.1038/s41467-020-14950-5)
Supplement: Supplementary file 3 — Reporting Summary [file 41467_2020_14950_MOESM3_ESM.pdf]

nature research

Corresponding author(s): Dominic van Essen
Simona Saccani

Last updated by author(s): Feb 4, 2020

Reporting Summary

Nature Research wishes to improve the reproducibility of the work that we publish. This form provides structure for consistency and transparency in reporting. For further information on Nature Research policies, see Authors & Referees and the Editorial Policy Checklist.

Statistics

For all statistical analyses, confirm that the following items are present in the figure legend, table legend, main text, or Methods section.

n/a	Confirmed	
☐	☒	The exact sample size (*n*) for each experimental group/condition, given as a discrete number and unit of measurement
☐	☒	A statement on whether measurements were taken from distinct samples or whether the same sample was measured repeatedly
☐	☒	The statistical test(s) used AND whether they are one- or two-sided<br>*Only common tests should be described solely by name; describe more complex techniques in the Methods section.*
☒	☐	A description of all covariates tested
☐	☒	A description of any assumptions or corrections, such as tests of normality and adjustment for multiple comparisons
☐	☒	A full description of the statistical parameters including central tendency (e.g. means) or other basic estimates (e.g. regression coefficient) AND variation (e.g. standard deviation) or associated estimates of uncertainty (e.g. confidence intervals)
☐	☒	For null hypothesis testing, the test statistic (e.g. *F*, *t*, *r*) with confidence intervals, effect sizes, degrees of freedom and *P* value noted<br>*Give P values as exact values whenever suitable.*
☒	☐	For Bayesian analysis, information on the choice of priors and Markov chain Monte Carlo settings
☒	☐	For hierarchical and complex designs, identification of the appropriate level for tests and full reporting of outcomes
☐	☒	Estimates of effect sizes (e.g. Cohen's *d*, Pearson's *r*), indicating how they were calculated

Our web collection on statistics for biologists contains articles on many of the points above.

Software and code

Policy information about availability of computer code

Data collection	genome alignment: bowtie v1.2; affymetrix data processing: 'affy' package, bioconductor v1.40.0
Data analysis	genomic dataset processing: bedtools v2.17.0; data processing: perl v5.20.1; data analysis: R v3.6.0

For manuscripts utilizing custom algorithms or software that are central to the research but not yet described in published literature, software must be made available to editors/reviewers. We strongly encourage code deposition in a community repository (e.g. GitHub). See the Nature Research guidelines for submitting code & software for further information.

Data

Policy information about availability of data

All manuscripts must include a data availability statement. This statement should provide the following information, where applicable:

- Accession codes, unique identifiers, or web links for publicly available datasets
- A list of figures that have associated raw data
- A description of any restrictions on data availability

All datasets generated in this study are available from the NCBI Gene Expression Omnibus (GEO) database with accession number GSE142170 . The source data underlying figures 1g, 2c,d, & 5a, and supplementary figures 2c-3, 3g & 9d, are provided as a source data file.

Field-specific reporting

Please select the one below that is the best fit for your research. If you are not sure, read the appropriate sections before making your selection.

☒ Life sciences ☐ Behavioural & social sciences ☐ Ecological, evolutionary & environmental sciences

Life sciences study design

All studies must disclose on these points even when the disclosure is negative.

Sample size	Sample biological replicates: DCs = 2 (non-stim) + 2 (stim); fibroblasts = 2 (non-stim) + 2 (stim) + 2 (non-stim, control for BAF-kd) + 2 (stim, control for BAF-kd) + 2 (non-stim, BAF-kd) + 2 (stim, BAF-kd) + 2 (stim, for pol-II S7P ChIP-MNase) + 2 (stim, for pol-II S5P ChIP-MNase).
Data exclusions	All replicates were included in analyses. Promoters with low-coverage ChIP-MNase data were excluded (12322 out of 25881).
Replication	Reproducibility between replicates and samples is detailed in figures 1e-g, supplementary figures 3g,16c,. Concordance with publicly-available data is detailed in supplementary figures 3a,b,8a-c.
Randomization	not applicable
Blinding	not applicable

Reporting for specific materials, systems and methods

We require information from authors about some types of materials, experimental systems and methods used in many studies. Here, indicate whether each material, system or method listed is relevant to your study. If you are not sure if a list item applies to your research, read the appropriate section before selecting a response.

Materials & experimental systems

n/a	Involved in the study	
☐	☒	Antibodies
☐	☒	Eukaryotic cell lines
☒	☐	Palaeontology
☒	☐	Animals and other organisms
☒	☐	Human research participants
☒	☐	Clinical data

Methods

n/a	Involved in the study	
☐	☒	ChIP-seq
☒	☐	Flow cytometry
☒	☐	MRI-based neuroimaging

Antibodies

Antibodies used	ntibodies used are detailed in the Methods section of the manuscript.
Validation	Commercial antibodies were validated by manufacturers. In addition, anti-H3K4me1, anti-p65, anti-pol-II S7P and anti-pol-II S5P were validated by analysis of ChIP-seq profiles (supplementary figures 1c,14,16a).

Eukaryotic cell lines

Policy information about cell lines

Cell line source(s)	Cell lines used are detailed in the Methods section of the manucript.
Authentication	None of the cell lines used were authenticated.
Mycoplasma contamination	All cell lines were tested negative for myclplasma contamination.
Commonly misidentified lines (See ICLAC register)	not applicable

ChIP-seq

Data deposition

☒ Confirm that both raw and final processed data have been deposited in a public database such as GEO.

☒ Confirm that you have deposited or provided access to graph files (e.g. BED files) for the called peaks.

Data access links *May remain private before publication.*	NCBI Gene Expression Omnibus (GEO) database accession number GSE142170 [https://www.ncbi.nlm.nih.gov/geo/query/acc.cgi?&acc=GSE142170]
Files in database submission	GSM4219551

Files in database submission

GSM4219552
GSM4219553
GSM4219554
GSM4219555
GSM4219556
GSM4219557
GSM4219558
GSM4219559
GSM4221916
GSM4221917
GSM4221918
GSM4221919
GSM4221920
GSM4221921
GSM4221922
GSM4221923
GSM4221924
GSM4221925
GSM4221926
GSM4221927
GSM4221928
GSM4221929
GSM4221930

Genome browser session (e.g. UCSC)

no longer applicable

Methodology

Replicates

Sample biological replicates: DCs = 2 (non-stim) + 2 (stim); fibroblasts = 2 (non-stim) + 2 (stim) + 2 (non-stim, control for BAF-kd) + 2 (stim, control for BAF-kd) + 2 (non-stim, BAF-kd) + 2 (stim, BAF-kd) + 2 (stim, for pol-II S7P ChIP-MNase) + 2 (stim, for pol-II S5P ChIP-MNase).

Sequencing depth

Sequencing depths for each replicate sample were between 20M and 50M reads (or read-pairs). H3K4me1 ChIP-MNase samples were sequenced using single-end 35bp or 50bp reads; pol-II S7P and S5P ChIP-MNase samples were sequenced using paired-end 50+35bp reads.

Antibodies

Antibodies used are detailed in the Methods section of the manuscript.

Peak calling parameters

Genome alignment was performed using bowtie options -v 2 -a -m 5 --tryhard for single-end sequencing, or -v 2 -a -m 5 --maxins 2000 --tryhard for paired-end sequencing

Data quality

not applicable

Software

genomic dataset processing: bedtools v2.17.0; data processing: perl v5.20.1; data analysis: R v3.6.0
